# Comparative Genomics of Multiple Strains of *Pseudomonas cannabina* pv. *alisalensis*, a Potential Model Pathogen of Both Monocots and Dicots

**DOI:** 10.1371/journal.pone.0059366

**Published:** 2013-03-28

**Authors:** Panagiotis F. Sarris, Emmanouil A. Trantas, David A. Baltrus, Carolee T. Bull, William Patrick Wechter, Shuangchun Yan, Filippos Ververidis, Nalvo F. Almeida, Corbin D. Jones, Jeffery L. Dangl, Nickolas J. Panopoulos, Boris A. Vinatzer, Dimitrios E. Goumas

**Affiliations:** 1 Department of Plant Sciences, School of Agricultural Technology, Technological Educational Institute of Crete, Heraklion, Greece; 2 Department of Biology, University of Crete, Heraklion, Greece; 3 School of Plant Sciences, The University of Arizona, Tucson, Arizona, United States of America; 4 United States Department of Agriculture–Agricultural Research Service, Salinas, California, United States of America; 5 United States Department of Agriculture–Agricultural Research Service, Charleston, South Carolina, United States of America; 6 Department of Plant Pathology, Physiology, and Weed Science, Virginia Tech, Blacksburg, Virginia, United States of America; 7 School of Computing, Federal University of Mato Grosso do Sul, Mato Grosso do Sul, Brazil; 8 Howard Hughes Medical Institute and Department of Biology, University of North Carolina at Chapel Hill, Chapel Hill, North Carolina, United States of America; 9 Carolina Center for Genome Sciences, University of North Carolina at Chapel Hill, Chapel Hill, North Carolina, United States of America; 10 Professor Emeritus, University of Crete, Heraklion, Greece and University of California, Berkeley, California, United States of America; Centre National de la Recherche Scientifique, Aix-Marseille Université, France

## Abstract

Comparative genomics of closely related pathogens that differ in host range can provide insights into mechanisms of host-pathogen interactions and host adaptation. Furthermore, sequencing of multiple strains with the same host range reveals information concerning pathogen diversity and the molecular basis of virulence. Here we present a comparative analysis of draft genome sequences for four strains of *Pseudomonas cannabina* pathovar *alisalensis* (*Pcal*), which is pathogenic on a range of monocotyledonous and dicotyledonous plants. These draft genome sequences provide a foundation for understanding host range evolution across the monocot-dicot divide. Like other phytopathogenic pseudomonads, *Pcal* strains harboured a *hrp/hrc* gene cluster that codes for a type III secretion system. Phylogenetic analysis based on the *hrp/hrc* cluster genes/proteins, suggests localized recombination and functional divergence within the *hrp/hrc* cluster. Despite significant conservation of overall genetic content across *Pcal* genomes, comparison of type III effector repertoires reinforced previous molecular data suggesting the existence of two distinct lineages within this pathovar. Furthermore, all *Pcal* strains analyzed harbored two distinct genomic islands predicted to code for type VI secretion systems (T6SSs). While one of these systems was orthologous to known *P. syringae* T6SSs, the other more closely resembled a T6SS found within *P. aeruginosa*. In summary, our study provides a foundation to unravel *Pcal* adaptation to both monocot and dicot hosts and provides genetic insights into the mechanisms underlying pathogenicity.

## Introduction

*Pseudomonas cannabina* is a Gram-negative, fluorescent, flagellated, aerobic bacterium that causes leaf and stem spot of hemp (*Cannabis sativa*), from which it derives its species name [1]. Although it was classified as a pathovar of *Pseudomonas syringae*, following extensive polyphasic taxonomic studies (including DNA-DNA hybridization, carbon source utilization and ribotyping) this pathovar was revived as a species in 1999 (*P. cannabina* pv. *cannabina*; [2]).

*Pseudomonas syringae* pv. *alisalensis* was recently transferred to *Pseudomonas cannabina* [3]. *P. cannabina* pv. *alisalensis* strains are not pathogenic on *C. sativa* but are frequently identified as the causal agent of bacterial blight diseases of field and greenhouse grown crucifers. Reciprocally, *P. cannabina* pv. *cannabina* strains are not pathogenic on crucifers. These differences in host range demonstrated that *P. cannabina* consists of at least two distinct pathovars, *P. cannabina* pv. *cannabina* (*Pcan*), and *P. cannabina* pv. *alisalensis* (*Pcal*) [3]. In addition to host range differences, *P. cannabina* pv. *alisalensis* also physiologically differs from *P. cannabina* pv. *cannabina* isolates with regards to carbon source utilization (Manitol, D(+) arabitol, Myoinositol, D-mannitol, D(-) tartrate), bacteriophage sensitivity, and pigment production further supporting distinct pathovars in *P. cannabina* [3].

Plant pathologists have only differentiated crucifer diseases caused by *P. syringae* pv. *maculicola* (*Pma*) and *Pcal* since 2000, thus some isolates from crucifers identified as *Pma* prior to 2000 were actually *Pcal* [3], [4]. For example, the well-studied *P. syringae* pv. *maculicola* strain B70 (otherwise known as CFBP 1637, M4, ES4326, and ICMP 4326), isolated from diseased radish in Wisconsin by Williams and Keen in 1965 [5] was recently shown to belong to *P. cannabina* pv. *alisalensis* using Multi-Locus Sequence Analysis (MLSA) [3]. *Pcal* ES4326 has been used extensively in various studies of plant-bacteria interactions and is pathogenic on several ecotypes of the model plant *Arabidopsis thaliana* [6]–[9].

*Pcal* has been isolated from diseased crucifers (e.g. arugula, *Eruca sativa* [10]–[12]; cabbage, *Brassica oleracea* var. *capitata* [13]; cauliflower, *Brassica oleracea* subsp. *botrytis* [14]; Brussels sprouts, *Brassica oleracea* subsp. *gemmifera* [3], [15]; rape, *Brassica napus* [4]; white mustard, *Brassica hirta* [4]; rutabaga, *Brassica napus* var. *napobrassica* [16] and radish, *Raphanus sativus* [3], [17]). However, *Pcal* can infect a wider range of hosts under experimental conditions including dicots (*Solanum lycopersicum* [10], [18]) as well as monocots (e.g. California brome, *Bromus carinatus*; oat, *Avena sativa*; common timothy, *Phleum pretense* and *Bromus diandrus* [3], [10], [18]). Thus, *P. cannabina* has the potential to be a model for studying plant-bacterial interactions across divergent hosts and can serve as a parallel model system to *P. syringae* for understanding virulence and host range evolution.

The Type III Secretion System (T3SS) is an essential mediator of the interaction of bacterial phytopathogens with their plant hosts and shares a common ancestor with the bacterial flagellum [19]–[22]. The T3SS is an inter-kingdom transfer device, which translocates a diverse array of proteins, called Type III Effector Proteins (T3EPs), through the type III pilus, either to extracellular locations or directly into the plant cells. T3EPs modulate host immune responses and determine the outcome of host-pathogen interactions [20]. *Pcal* strain ES4326 was the first strain of any plant pathogenic *Pseudomonas* species for which the majority of the T3EP repertoire was experimentally determined [9]. However, little is known about the conservation of T3EPs and other pathogenicity factors like the type VI secretion system (T6SS) and toxins throughout *Pcal*. The T6SS is a recently discovered protein secretion/translocation system found in a large number of bacteria, including phytopathogenic and plant-associated bacteria [23], [24]. Although T6SSs are widespread, and are often present in multiple copies in the genomes of phytopathogens, little is known about the exact role of this system in plant-bacterial interactions. The T6SS has been studied primarily in the context of mammalian pathogenic bacteria-host interactions and may also function to promote commensal or mutualistic relationships between bacteria and eukaryotes, as well as to mediate cooperative or competitive interactions between bacterial species [24]. In a recent work, Haapalainen and colleagues [25] present evidence for the requirement of one of the *P. syringae* T6SSs, for the survival in competition with enterobacteria and yeasts, through growth suppression of these competitors.

Comparative genomics can provide insights into host-pathogen interactions, differences in virulence factor repertoires, and pathogen evolution [26]. Several, complete or draft genome sequences have been generated for important phytopathogenic pseudomonads, including: *P. syringae* pv. *tomato* (*Pst*; [27], [28]), *P. syringae* pv. *phaseolicola* (*Psph*; [29]), *P. syringae* pv. *syringae* (*Pss* B728a [30], *Ps* FF5 [31]), *P. syringae* pv. *oryzae* (*Psor*; [32]), among others [33]–[37]. Baltrus and colleagues reported the analysis of multiple genomes within *P. syringae,* and including *Pcal* ES4326 (although this strain was referred to as *P. syringae* pv. *maculicola*), to reveal the genetic changes underlying differences in host range and virulence across host plants ranging from rice to maple trees [37].

The wide host range of *Pcal* provides a unique opportunity to understand virulence evolution in a closely related parallel model system to *P. syringae*. For this purpose we selected to sequence the genomes of four representative strains from geographically distant areas; the USA strains *Pcal* BS91 (CFBP 6866 pathotype strain) [3], and *Pcal* T3C (CFBP 7684) [37], and the Greek strains *Pcal* PSa1_3 (CFBP 7682) [10], and *Pcal* PSa866 (CFBP 7683).

The comparison of (a) the sequenced *Pcal* genomes with each other, and (b) with the genomes of other related pathogens, will help us to better understand the structure and function of the genomic elements that give each strain its unique virulence characteristics. More specifically, given their demonstrated importance for inter-organismal interactions, we focused our analysis on the genes coding for the T3SS, T6SSs, and T3EPs. Where possible, we also compared our findings with a draft genome sequence of the *Pcan* type strain (CFBP 2341 [2]), despite the poor quality of the *Pcan* genome sequence; and the available data from the *Pcal* ES4326 genome [37], [38]. Our analysis demonstrated intra-pathovar variation in T3EPs content and phylogeny revealing two distinct lineages within *Pcal*, in agreement with previously reported rep-PCR and MLSA results [3]. The data presented here will provide the basis for focused molecular plant-microbe investigations into patho-adaptation of *Pcal* strains to plants and other inter-organismal interactions involving *Pcal*.

## Results and Discussion

### Pathogenicity Tests and Determination of Host Range

**Figure 1:**
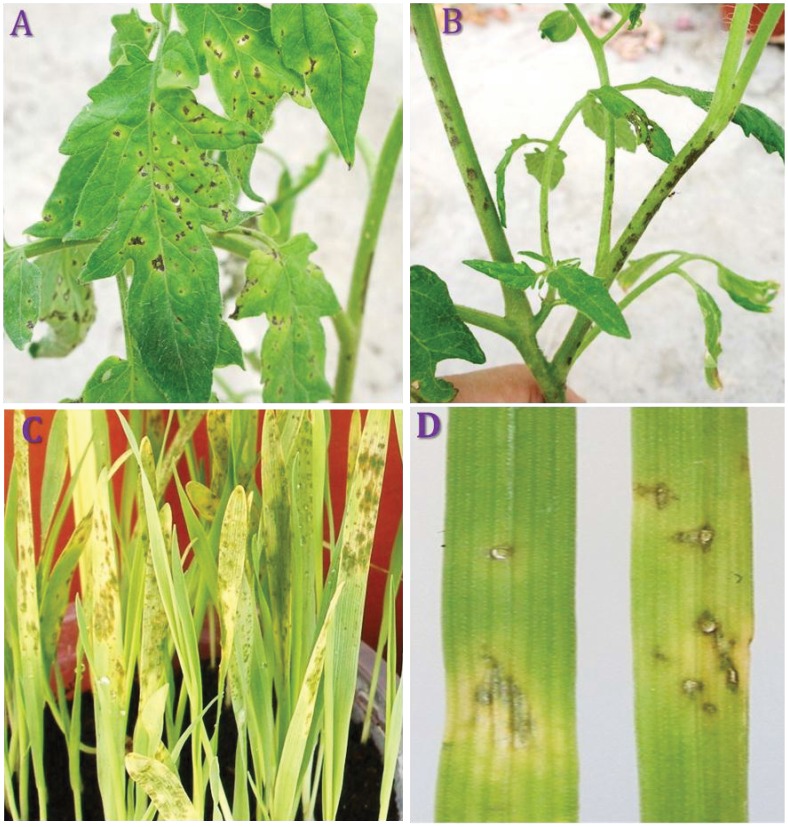
Compatible reactions to artificial inoculations of *P. cannabina* pv. *alisalensis* (*Pcal*) on monocots and dicots plant species. Artificial inoculations were performed using the sequenced *Pcal* strain PSa1_3 on: tomato leaves (**A**), tomato stem (**B**), Oat leaves (**C**) and *Bromus diandrus* leaves (**D**). Information for additional artificial inoculations on various plant species can be found in figure S1.

A range of hosts to which the *Pcal* strains BS91, T3C and ES4326 are pathogenic was previously determined [3], [18], [39]. We found that the *Pcal* strains PSa1_3 and PSa866 had identical experimentally determined host ranges (Figure 1 and Figure S1). Disease symptoms occurred after inoculation of the dicots *Eruca sativa*; *Brassica oleracea* var. *capitata*, *Brassica oleracea* subsp. *botrytis*, *Brassica oleracea* subsp. *gemmifera*, *Brassica napus*, *Brassica hirta*, *Brassica napus* var. *napobrassica* and *Raphanus sativus* and *Solanum lycopersicum,* as well as the monocots *Avena sativa* and *Bromus diandrus* with all *Pcal* strains. All strains induced the same disease symptoms regardless of their original host of isolation, which argues against extensive specialization within *Pcal* across the tested hosts (Figure 1 and Figure S1).

### Genome-wide Sequence Data

A draft genome sequence for *Pcal* ES4326 was recently published [37]. To characterize diversity within *Pcal* and to compare virulence gene repertoires within *Pcal* and with *P. syringae* pathovars, we sequenced the genomes of PSa1_3, PSa866, BS91 and T3C. Strains PSa1_3 and PSa866 were sequenced on a 314 chip of the ION Torrent PGM (Institute of Molecular Biology and Biotechnology, Greece) while strains BS91 and T3C were sequenced on an Illumina GAII (University of North Carolina, USA).

For the *Pcal* PSa1_3 strain a total of 655.105 DNA fragments of an average length of 107 nts were obtained, totaling 70.166.144 nts. The reads were assembled into 489 contigs yielding a total draft genome size of 6.022.892 nts and representing a genome coverage of 11,6 x. For the *Pcal* PSa866 strain a total of 547.652 DNA fragments of an average length of 77 nts were read, totaling 42.249.079. The reads were assembled in 777 contigs yielding a total draft genome size of 5.849.615 nts and representing a genome coverage of 7,2x (Table 1).

**Table 1:** Statistics and features of the sequenced *P. cannabina* pv. *alisalensis* strains.

Pcal strain	PSa1_3		PSa866		ES4326		BS91		T3C	
Origin and Reference	Greece [10]		Greece [10]		USA [5]		USA [18]		USA [39]	
Taxon ID	2.512.564.082		2.512.564.081		2.505.313.069		2.516.653.056		2.516.653.057	
Total output	70.166.144		42.249.079		524.136.480		453.847.450		259.720.930	
Number of reads	655.105		547.652		14.975.328		12.967.070		7.420.598	
Average read length	107		77		35		35		35	
Sequence coverage	11,6x		7,2x		84,2x		75x		33x	
DNA scaffolds	489		777		878		381		464	
	**Number**	**% of Total**	**Number**	**% of Total**	**Number**	**% of Total**	**Number**	**% of Total**	**Number**	**% of Total**
DNA, total number of bases	6.022.892	100%	5.849.615	100%	6.221.751	100%	6.039.137	100%	5.821.618	100%
Protein coding number of bases	5.189.942	80,13%	4.885.192	83,51%	5.470.461	87,92%	5.324.805	88,17%	5.129.791	88,12%
DNA G+C number of bases	3.534.627	58,69%	3.420.373	58,48%	3.637.285	58,46%	3.547.884	58,75%	3.419.407	58,74%
Genes total number	6.930	100%	8.436	100%	6.266	100%	5.721	100%	5.630	100%
Protein coding genes	6.829	98,54%	8.354	99,03%	6.205	99,03%	5.617	98,18%	5.528	98,19%
Protein coding genes with function prediction	4.883	70,46%	5.166	61,24%	4.504	71,88%	4.311	75,35%	4.255	75,58%
Protein coding genes without function prediction	1.946	28,08%	3.188	37,79%	1.701	27,15%	1.306	22,83%	1.273	22,61%
Protein coding genes with homology to functionally characterized enzymes	982	14,17%	642	7,61%	1.197	19,10%	1.225	21,41%	1.193	21,19%
Protein coding genes with homology only to candidate KO based enzymes	622	8,98%	1.387	16,44%	137	2,19%	43	0,75%	75	1,33%
Fused Protein coding genes	13	0,19%	5	0,06%	61	0,97%	13	0,23%	12	0,21%
Genes carrying signal peptides	1.192	17,20%	1.203	14,26%	1.255	20,03%	1.178	20,59%	1.153	20,48%
Genes coding trans-membrane proteins	1.316	18,99%	1.438	17,05%	1.263	20,16%	1.142	19,96%	1.104	19,61%

The ES4326 strain’s genome features have earlier been reported by Baltrus et al. [37].

Illumina sequencing of *Pcal* BS91 gave an output of 12.967.070 paired-end sequences of an average length of 2×35 nts, totaling 453.847.450 nts. Reads were assembled into 381 scaffolds yielding a draft genome with size of 6.039.137 nts (75×coverage). The sequencing of the *Pcal* T3C strain gave 7.420.598 reads of an average length of 35 nts, totaling 259.720.930 nts. These reads were assembled into 464 scaffolds yielding a draft genome with the size of 5.821.618 nts (33×coverage).

Genome size for the four strains was within the range of previously sequenced and published *P. syringae* draft genomes [32]–[37]. Also the G+C content (Table 1) was similar to sequenced *P. syringae* genomes. Sequence assemblies from all *Pcal* strains have been deposited to JGI/IMG-ER under the following accessions: BS91 (project ID 28.208, Taxon OID 2516653056), T3C (project ID 28211, Taxon OID 2516653057), PSa1_3 (project ID 6398, Taxon OID 2512564082), PSa866 (project ID 6397, Taxon OID 2512564081).

### Phylogenetic Relationship and Protein Comparison between *Pcal* and the *P. syringae* Species Complex

To determine the phylogenetic relationship and differences in protein repertoires between the sequenced *Pcal* strains, genomes of representative strains of the *P. syringae* species complex (Table S3) were compared with the annotated *Pcal* genomes. Results of the protein comparison can be viewed at this URL: http://pacu.facom.ufms.br/Pcal/.

Prediction of protein repertoires is limited by the draft status of most pseudomonad genome sequences so that predicted proteins encoded by genes at the end of contigs may be missing their C-termini or N-termini and may have a higher percentage of sequencing errors due to lower sequencing depth compared to those found in the middle of contigs. However, considering these limitations, there are 710 predicted protein families with members (orthologs and in-paralogs) in each *Pcal* and each *P. syringae* genome sequence and 50 predicted protein families that have members (orthologs and in-paralogs) present in each of the five *Pcal* genome but absent from all sequenced *P. syringae* genomes. Although some are short hypothetical proteins, approximately 30 proteins have predicted functions and may be indicative of *Pcal*-specific metabolism and/or virulence mechanisms, for example, a predicted carbamoyl transferase of the NodU family, two predicted acyltransferases, a predicted beta-lactamase, and a multidrug efflux system transmembrane protein (possibly conferring antibiotic and/or plant toxin resistance).

**Figure 2:**
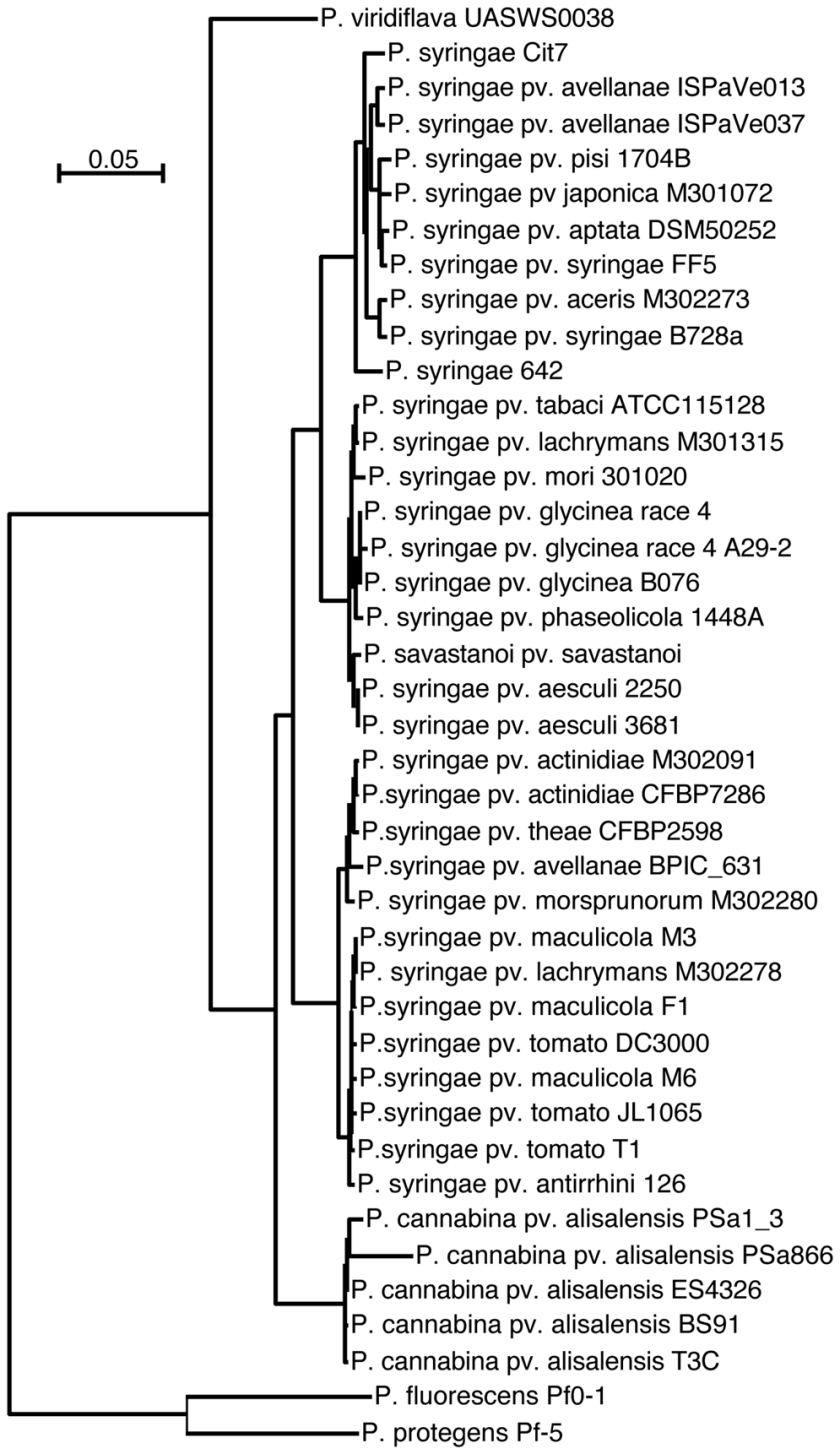
Phylogenetic tree of *P. cannabina* pv. *alisalensis* (*Pcal*) and the *P. syringae* species complex based on core genome proteins. A phylogenetic tree was constructed based on all proteins present exactly one time in each of the *Pcal* genome sequences and representative *P. syringae* genome sequences. The Bootstrap values for all branches of the tree are 100. The genomes and their accession numbers are listed in Table S3.

All proteins present exactly one time in each genome of the *P. syringae* species complex (including the *Pcal* genomes) were aligned and used to construct a phylogenetic tree (Figure 2). It can be clearly seen that *Pcal* strains form a separate clade compared to the rest of the *P. syringae* species complex but still share a more recent common ancestor with *P. syringae* than with *P. viridiflava*. *P. fluorescens* is the *Pseudomonas* species most closely related to the genome-sequenced *P. syringae* and *P. viridiflava* strains. *P. fluorescens* strains were thus used as outgroup.

Within the *Pcal* clade, strain PSa866 appears to have diverged significantly from the rest of *Pcal*. However, since the genome sequence of PSa866 has the lowest relative genome coverage and the PSa866 assembly is more fragmented than the other *Pcal* genome sequences, this apparent divergence may be the result of sequencing errors.

### Alignment between the Draft Genomes of *Pcal* and the Complete Genomes of *Pss* B728a and *Pst* DC3000

**Figure 3:**
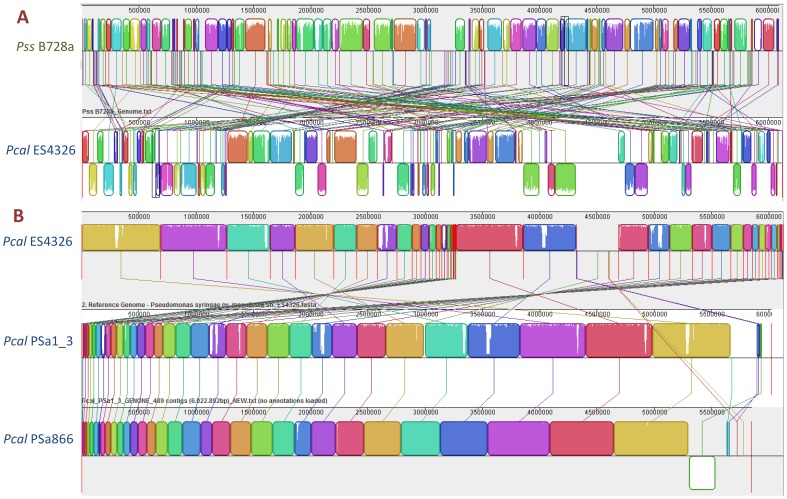
Genome alignments. Pairwise alignment between *Pcal* ES4326 (previously known as *P. s.* pv. *maculicola* ES4326) and the compete genome of *P. s.* pv. *syringae* B728a (**A**) and the draft genomes of Lineage-I *Pcal* PSa866 and Lineage-II *Pcal* PSa1_3 (**B**) using the MAUVE software. Colored blocks outline genome sequence that aligned to part of another genome, and was presumably homologous and internally free from genomic rearrangement (Locally Colinear Blocks or LCBs). White regions are sequence that were not aligned and probably contain sequence elements specific to a particular genome. Blocks below the center line indicate regions that aligned in the reverse complement (inverse) orientation. The height of the profile within each LCB demonstrates the average degree of sequence conservation within an aligned region.

We investigated the overall genomic differences between *Pcal* and *Pss* B728a and *Pst* DC3000 by aligning the previously sequenced *Pcal* ES4326 genome and the *Pcal*, PSa1_3 and PSa866 genomes sequenced here with the closed genomes of *Pss* B728a and *Pst* DC3000 using MAUVE 2.3.1 software [40] (Figure 3 and Figure S2). The *Pcal* PSa1_3 and PSa866 genomes were highly syntenic with *Pcal* ES4326 confirming their close relationship (Figure 3B). The alignments between: a) *Pcal* ES4326 and *Pss* B728a (Figure 3A), b) *Pcal* PSa1_3, *Pcal* PSa866 and *Pcal* ES4326 (Figure 3B), and c) *Pcal* ES4326, *Pcal* PSa1_3 and *Pcal* PSa866 and the complete genome of *Pst* strain DC3000 (Figure S2), are also presented. Since all *Pcal* genomes are unfinished, only genomic rearrangement that occurred within contigs are identifiable and the actual number of rearrangements may therefore be higher.

### General Features of the *Pcal* Draft Genome Sequences Compared to *Pcal* ES4326

Table 1 lists genome features for the sequenced *Pcal* strains. Alignments of the draft genome assemblies for *Pcal* PSa1_3, *Pcal* PSa866 and *Pcal* ES4326 suggested a high level of conservation across the chromosome. The first two strains had highly syntenic chromosomes, but many syntenic blocks between them were presented on different relative positions in the ES4326 genome (Figure 3B). Based on the total length of *Pcal* assemblies, as well as the size of the *Pcal* ES4326 draft genome (6.221.751 bp), we estimate the depths of coverage and that the 96,8% of *Pcal* PSa1_3 genome and 94,1% of *Pcal* PSa866 was represented in their respective draft genomes.

**Figure 4:**
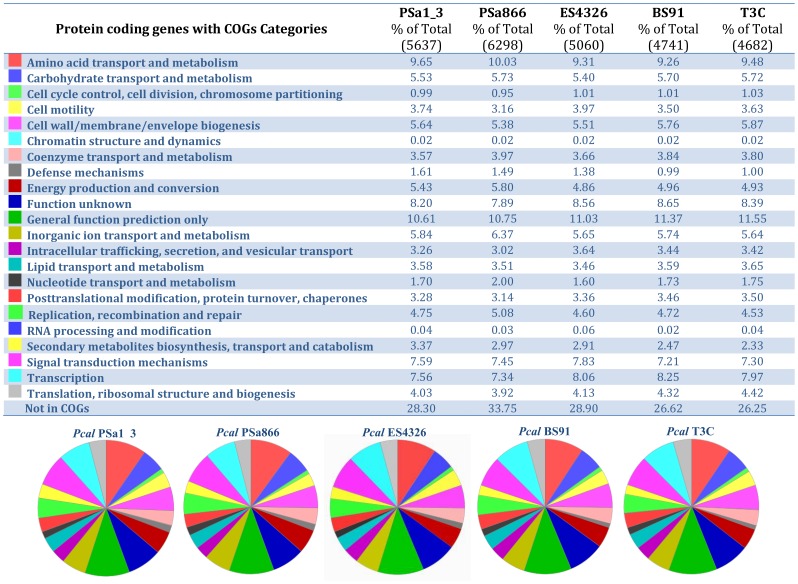
Presentation of the distribution of various gene categories (COGs) in the respective *Pcal* strains PSa1_3, PSa866, BS91, T3C and ES4326.

Comparison of these *Pcal* draft genomes to other publically available assemblies, revealed significant heterogeneity for several genomic parameters. Several differences between the *Pcal* genomes were found in regard to: a) prediction of protein coding genes without function; b) enzyme-encoding genes; c) protein coding genes without assigned enzymatic function, but with similarity to groups of enzymes based on KEGG Orthology (KO); and d) the predicted fusions of protein coding genes (Figure 4; Table 1). However, because of the draft status of the assembled genomes it is unclear whether these differences represented true differences between strains or they were sequencing or assembly artifacts.

In an attempt to predict and assemble the plasmids harbored by the strains under investigation, reference assemblies utilizing publicly available plasmid sequences from strain ES4326 were used [38]. *Pcal* ES4326 contained five plasmids: pPCAL4326A (46.697 bp), pPMA4326B (40.110 bp), pPMA4326C (8.244 bp), pPMA4326D (4.833 bp) and pPMA4326E (4.217 bp). We found that sequences showing homology to regions of plasmids pPMA4326A and pPMA4326B were present in all *Pcal* strains, but no evidence was found for the presence of pPMA4326C, pPMA4326D and pPMA4326E in any of them. Surprisingly, the *Pcan* BS0968 strain harbor sequences showing homology to regions of three of the *Pcal* ES4326 plasmids (pPMA4326A, pPMA4326B and pPMA4326C) but not pPMA4326D and pPMA4326E (Table S2).

### Secretion Systems in *P. cannabina* pv. *alisalensis*

All five *Pcal* strains (PSa1_3, PSa866, BS91, T3C and ES4326) possessed loci with similarity to structural genes involved in the biogenesis of the type I, II, III and VI secretion systems. Genes for Autotransporter-1 (AT-1) family proteins of the type V secretion system, the general secretion (Sec) pathway, and the twin-arginine translocation (Tat) pathway were also present in all five *Pcal* strains. All of the above mentioned secretion systems are present in several other sequenced plant pathogenic pseudomonads, with the exception of the T1SS, which is absent in the genome of *Pst* DC3000 [41], [42].

Complete *hrp/hrc* gene clusters encoding T3SSs of the “Hrp-1 family” [43] were also found in all five *Pcal* strains (GenBank Accession N° of *Pcal* PSa1_3 T3SS: JQ517282) and closely resembled the *hrc/hrp* cluster of *P. syringae* B728a. Strains *Pcal* PSa1_3 and *Pcan* BS0968 harbored a complete T4SS (classified as a “P” group T4SS according to Souza et al. [44]) as was previously reported for strain *Pcal* ES4326 (located on plasmid pPMA4326A [37]). However, only a portion of this T4SS was identified in the *Pcal* PSa866 genome. Surprisingly, the other two *Pcal* strains (T3C and BS91) did not appear to carry genes related to this system, even though all three appear to harbor a plasmid similar to pPMA4326A of *Pcal* ES4326.

Each of the five *Pcal* genomes contained two clusters coding for predicted T6SSs (GenBank Accession N° for *Pcal* PSa1_3 T6SS-I: JQ517283 and for *Pcal* PSa1_3 T6SS-II: JQ517284) [24]. The functionality of these two T6SSs and their exact role during pathogenesis or other aspects of *Pcal* biology has yet to be investigated. Interestingly, recently a role for the *Pst* DC3000 T6SS and the T6SS cargo protein Hcp2 in *P*s*t* survival in competition with enterobacteria and yeasts was reported [25].

### Organization of the *hrp/hrc* Cluster in *P. cannabina* pv. *alisalensis*

**Figure 5:**
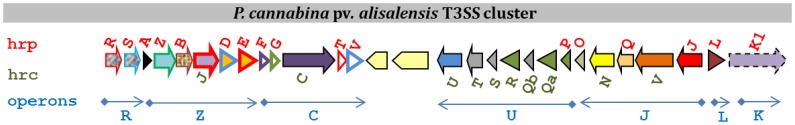
Schematic representation of the *hrp/hrc* gene *cluster* coding for the synthesis of the type III secretion system of *Pseudomonas cannabina* pv. *alisalensis*. The genes were arranged in five operons organized in two main blocks having convergent transcription: the *hrpRS*, *hrpZ* and *hrpC* operons in one orientation, and the *hrpU* and *hrpJ* in the opposite direction. The two blocks were separated by a hyper variable region with a very low level of conservation between closely taxonomically related bacteria.

The *hrp/hrc* clusters in all five examined *Pcal* genomes (PSa1_3, PSa866, BS91, T3C, and ES4326) were identical in size and orientation (Figure 5). The cluster spans 25,658 bp, and is composed of twenty-eight genes. In most of the *Pcal* strains T3SS genes were arranged in five operons organized in two main blocks having convergent transcription: the *hrpRS*, *hrpZ* and *hrpC* transcriptional units in one orientation, and the *hrpU* and *hrpJ* transcriptional units in the opposite direction. The two *Pcal* T3SS gene blocks were separated by a hyper variable region that was previously shown to have very low level of conservation even between taxonomically closely related bacteria [45]. In the *Pcal* genomes, this hyper variable region harbors two ORFs with unknown function, which were also present in *Pss* B728a (Psyr_1204, Psyr_1203) and in *P. syringae* pv. *aceris* str. M302273 (PSYAR_01264, PSYAR_01274) but are absent from all other sequenced *P. syringae* genomes. The two genes were located upstream of the *hrp/hrc* cluster (near *hrpL* and *hrpK*) and were not part of any operon.

This kind of organization has also been reported for other *P. syringae* pathovars harboring the “Hrp-1 family” *hrp/hrc* T3SS [20].

The G+C content of the entire T3SS cluster for all five *Pcal* strains (57.9%) was in the range reported for other *hrp/hrc* clusters of phylogenetically related *P. syringae* pathovars (56–58% [46]). The T3SS cluster of *Pcal* strains appears to be syntenic with all other phytopathogenic *P. syringae* strains, and appears chromosomally located and present in a single copy as assessed by BlastN searches. Our analysis also revealed thirty three Single Nucleotide Polymorphisms (SNPs) between the *Pcal* PSa1_3, PSa866 and T3C *hrp/hrc* clusters (Table S1). Identity between predicted Hrp/Hrc proteins from *Pcal* and those from *Pss* B728a, *Pst* DC3000 and *Psph* 1448a range from 46% (for HrpA of *Pst* DC3000) to 97% (for HrpF, hrpT, and HrpV of *Pss* B728a) (Table 2). Of note, most *Pcal* Hrp/Hrc proteins shared highest identity with those of *Pss* B728a, which further supported the close relationship between the T3SSs of *Pcal* and *Pss* B728a. This is surprising because the *Pss* B728a strain is more distantly related to the *Pcal* strains compared to other sequenced *P. syringae* strains.

**Table 2:** Aminoacid sequence identity percentages between predicted Hrp/Hrc proteins from *P. cannabina* pv. *alisalensis* and those from *P. syringae* pv. *syringae* B728a, *P. syringae* pv. *tomato* DC3000 and *P. syringae* pv. *phaseolicola* 1448a.

Pcal	Pss_B728a	Pst_DC3000	Psph_1448a
HrpR	95% Psyr_1190	94% PSPTO_1379	95% PSPPH_1270
HrpS	93% Psyr_1191	93% PSPTO_1380	94% PSPPH_1271
HrpA	63% Psyr_1192	46% PSPTO_1381	64% PSPPH_1272
HrpZ	82% Psyr_1193	66% PSPTO_1382	80% PSPPH_1273
HrpB	92% Psyr_1194	65% PSPTO_1383	89% PSPPH_1274
HrcJ	96% Psyr_1195	95% PSPTO_1384	96% PSPPH_1275
HrpD	89% Psyr_1196	82% PSPTO_1385	86% PSPPH_1276
HrpE	92% Psyr_1197	87% PSPTO_1386	91% PSPPH_1277
HrpF	97% Psyr_1198	64% PSPTO_1387	95% PSPPH_1278
HrpG	95% Psyr_1199	85% PSPTO_1388	90% PSPPH_1279
HrcC	95% Psyr_1200	91% PSPTO_1389	94% PSPPH_1280
HrpT	97% Psyr_1201	93% PSPTO_1390	99% PSPPH_5227
HrpV	97% Psyr_1202	90% PSPTO_1391	95% PSPPH_1281
Hyp. ORF1	88% Psyr_1203	NE	NE
Hyp. ORF2	94% Psyr_1204	NE	NE
HrcU	98% Psyr_1205	96% PSPTO_1392	98% PSPPH_1282
HrcT	99% Psyr_1206	93% PSPTO_1393	97% PSPPH_1283
HrcS	100% Psyr_1207	97% PSPTO_1394	99% PSPPH_1284
HrcR	100% Psyr_1208	99% PSPTO_1395	98% PSPPH_1285
HrcQb	84% Psyr_1209	77% PSPTO_1396	83% PSPPH_1286
HrcQa	86% Psyr_1210	82% PSPTO_1397	85% PSPPH_1287
HrpP	83% Psyr_1211	78% PSPTO_1398	79% PSPPH_1288
HrpO	92% Psyr_1212	91% PSPTO_1399	90% PSPPH_1289
HrcN	97% Psyr_1213	98% PSPTO_1400	96% PSPPH_1290
HrpQ	89% Psyr_1214	85% PSPTO_1401	87% PSPPH_1291
HrcV	98% Psyr_1215	97% PSPTO_1402	98% PSPPH_1292
HrpJ	82% Psyr_1216	78% PSPTO_1403	82% PSPPH_1293
HrpL	95% Psyr_1217	97% PSPTO_1404	96% PSPPH_1294
HrpK	86% Psyr_1218	82% PSPTO_1405	86% PSPPH_1295

For this analysis the hrp/hrc proteins of *P. cannabina* pv. *alisalensis* PSa1_3 were used. NE: do not exist.

### Phylogenetic Analysis of T3SS Clusters in *P. cannabina* pv. *alisalensis* Strains and Other Species of *P. syringae sensu lato*

**Figure 6:**
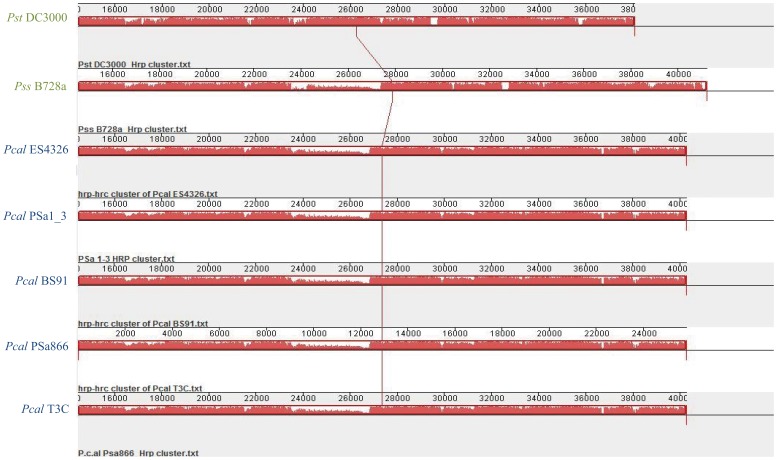
*hrp/hrc* clusters comparison. Pairwise alignment between the *hrp/hrc* gene clusters coding for the type III secretion systems of the complete sequenced *P. syringae* pv. *syringae* B728a and *P. syringae* pv. *tomato* DC3000 and those of *P. cannabina* pv. *alisalensis* PSa1_3, PSa866 and T3C. Areas where white is visible, were not aligned and probably contained sequence elements specific to a particular genome. The height of the profile demonstrates the average degree of sequence conservation within an aligned region.

**Figure 7:**
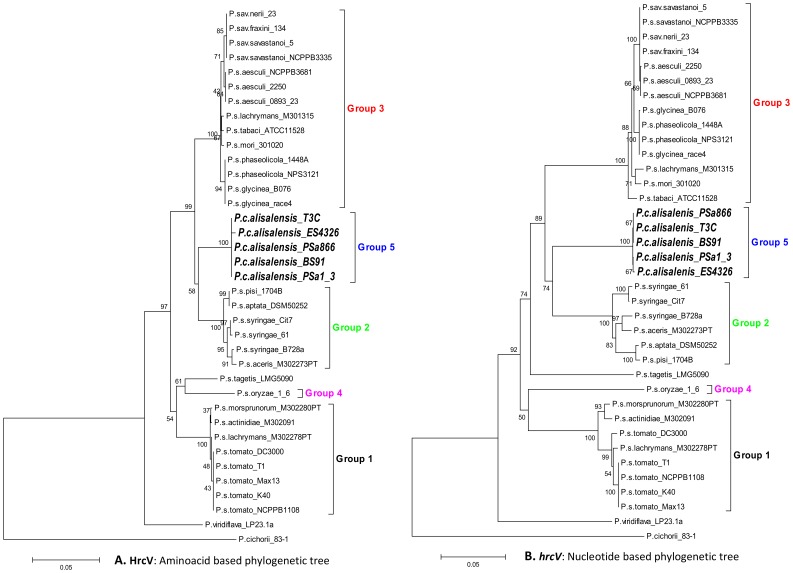
T3SS core component HrcV phylogenetic analysis. For the phylogenetic analysis the amino acid (**A**), as well as the nucleotide sequences (**B**), were used. Information for additional phylogenetic analysis of various T3SS core components can be found in figures S3. The various MLSA groups [43], [45] are marked in different colors.

We completed several phylogenetic analyses comparing several previously sequenced *P. syringae* isolates together with these five *Pcal* strains. The analysis included: a) comparison of the entire *hrp/hrc* cluster of *Pss* B728a, *Pst* DC3000 and the *hrp/hrc* cluster from the five *Pcal* strains (Figure 6) and b) phylogenetic analysis and comparison based on the nucleotide and amino acid sequences of the *hrcV* (Figure 7) and *hrpZ* and *hrcC* genes (Figure S3), which each represent an individual transcriptional unit of the T3SS gene cluster. The comparison of the entire T3SS cluster between investigated *Pcal* strains revealed significant conservation (Figure 6).

Phylogenetic characterization of *P. syringae* strains from different pathovars by Multilocus Sequence Analysis (MLSA) previously revealed five major groups of *P. syringae* [47], [48]. A recent study demonstrated that MLSA groups correspond to the nine genomospecies: genomospecies 1, 2, 3, 4 and 9 corresponds to MLSA groups 2, 3, 1, 4 and 5, respectively [47]. Members of genomospecies 6 and 8 are also distinct by MLSA but have not been given an MLSA group number. Likewise, studies based on the phylogenetic analysis of selected *hrp/hrc* genes investigated the relationship between *hrp*/*hrc* phylogenetic clustering and the MLSA grouping of *P. syringae* pathovars [43], [48], [49]. *Pcal* strain ES4326 clusters with MLSA group 5 (genomospecies 9), based on the concatenated DNA sequence of seven and four housekeeping genes, respectively [48], [49].

Our results based on the phylogenetic analysis of nucleotide as well as amino acid sequences of *hrcV* revealed nearly identical topologies. The *Pcal* T3SSs were grouped together and linked with *P. syringae* pathovars from the MLSA Group 2 (Figure 7; genomospecies 1). However, phylogenies based on *hrpZ* and *hrcC* amino acid and nucleotide sequences differed compared to *hrcV* (Figures 7 and S3). In both, *hrpZ* and *hrcC* trees, the *Pcal* strains clustered more closely with pv. *mori* and pv. *lachrymans* from MLSA Group 3 (genomospecies 2) with high bootstrap support (Figure S3). This unusual relationship among the MLSA groups 3 and 5 has been previously reported for the *hrpZ* operon and interpreted as a result of a putative recombination event [43]. Since our results revealed that the *hrcC* tree had the same basic topology as the *hrpZ* tree and since *hrcC* did not directly flank the *hrpZ* operon (Figure 5), we concluded that the recombination event similar to that inferred in [43] affected a considerably larger region than originally expected.

These data suggest that *P. syringae* and *Pcal* clades share a common ancestor that likely acquired the *hrp/hrc* cluster by a single Horizontal Genetic Transfer (HGT) event prior to their speciation [50], followed by localized HGT events within the T3SS.

Phylogenetic analysis also demonstrated that *hrpZ* and *hrpA* (Figure S3 and Table 2) were some of the most variable genes among the *P. syringae* and *Pcal* strains, confirming earlier reports from several *P. syringae* pathovars [46]. For example, the *Pst* DC3000 and *Pss* 61 *hrpA* genes are only 30% similar [46]. The genes coding for these two proteins are located in the *hrpZ* operon of the *hrp/hrc* cluster (Figure 5). Based on this fact, specific primers were designed to exploit this variability for diagnostic purposes in order to differentiate *P*. *syringae* strains at the pathovar level [51]. Based on our data (Table 2), the *Pcal hrpA* and *hrpZ* gene sequences would be useful targets for designing *Pcal*-specific primers.

### Distribution of Type III Effector Genes among *P. cannabina* pv. *alisalensis* Strains

**Figure 8:**
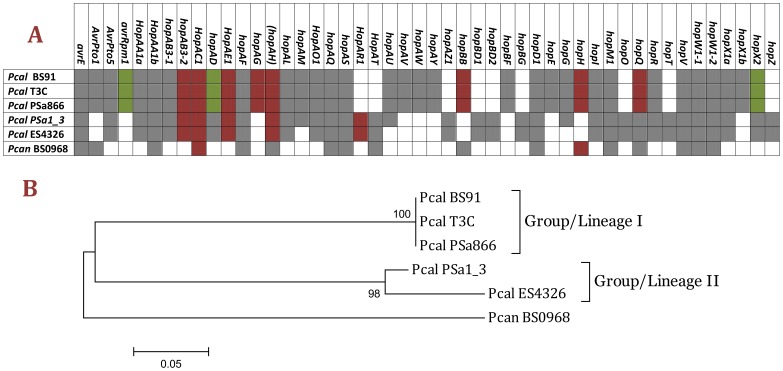
Comparison of the sequenced *Pcal* strains genomes, as well as of the *Pcal* ES4326 and *P. cannabina* pv. *cannabina* BS0968 genomes, for genes encoding candidate T3SS secreted substrates. In addition to known hop effector genes, two genes for T3SS helper proteins are also included (*hrpZ1* and *hrpW1*) (**A**). Occurrence of effector genes in the six strains is indicated: Absence (white), Presence (gray), Truncation (bordeaux), disrupted by a premature stop codon (green). Comparison tree contracted using the information presenting in the *presence-absence* table (part A of the same Figure) (**B**).

Using the Hop Database (HDB; http://www.pseudomonas-syringae.org), nucleotide and/or amino acid sequences of type III effectors (T3EPs) were used to screen the draft genome sequences of all five *Pcal* strains. This allowed us to identify the core T3EPs conserved in all five strains as well as strain-specific effectors (Figure 8A). A subset of 19 genes coding for T3EPs were found to form the core T3EP repertoire of *Pcal*: *avrE1, avrPto5, hopAA1a, hopAA1b, hopAB3-1, hopAF1, hopAL1, hopAO1, hopAQ, hopAS1, hopD1, hopI1, hopM1, hopR1, hopV1, hopW1-1, hopW1-2,* and *hopX1a* (Figure 8A). Conservation implied an important role for these T3EPs in pathogenicity of *Pcal*. A second set of genes coding for T3EPs (*avrRpm1, hopAB3-2, hopAC1, hopAD, hopAE1, hopAH1, hopQ1, HopX2,* and *hopAD1*) were present in all five strains but truncated or with an in-frame stop codon in at least one of the *Pcal* strains. A third subset of genes coding for T3EPs was strain-specific: *avrPto1, hopAM1, hopAT1, hopAU1, hopAV1, hopAW1, hopAY1, hopAZ1, hopBD1, hopBD2, hopBF1, hopBG, hopE1, hopG, hopO1, hopT1, hopX1b,* and *hopZ1*. Finally, there was a subset of truncated strain-specific effectors: *hopAG1, hopAR1, hopBB1,* and *hopH1* (Figure 8A).

*Pcal* strains formed two distinct lineages (Lineage-I and Lineage-II) according to their T3EP repertoires (Figure 8B), which mirrored the two lineages established within *Pcal* by rep-PCR and MLSA [3]. Over 150 strains in Lineage I, including the pathotype strain BS91, were isolated from brassica species [3], while all strains of Lineage-II (including PSa1_3 and ES4326) were isolated from radish or arugula. The only exception is one strain isolated from radish in Germany during an outbreak in 2008 that was attributed to Lineage-I based on rep-PCR [17]. All sequenced strains in Lineage-I (PSa866, T3C and BS91) had identical T3EP repertoires. However, there were some differences in the genes coding for T3EPs between strains of Lineage-II (ES4326 and PSa1_3), for example, *hopAM1*, *hopAU1*, *hopG1* were present in PSa1_3 but not in ES4326. Future studies will focus on whether these differences affect virulence and host range.

### Phylogenetic Relationships of *P. cannabina* pv. *alisalensis* Type III Effectors

**Figure 9:**
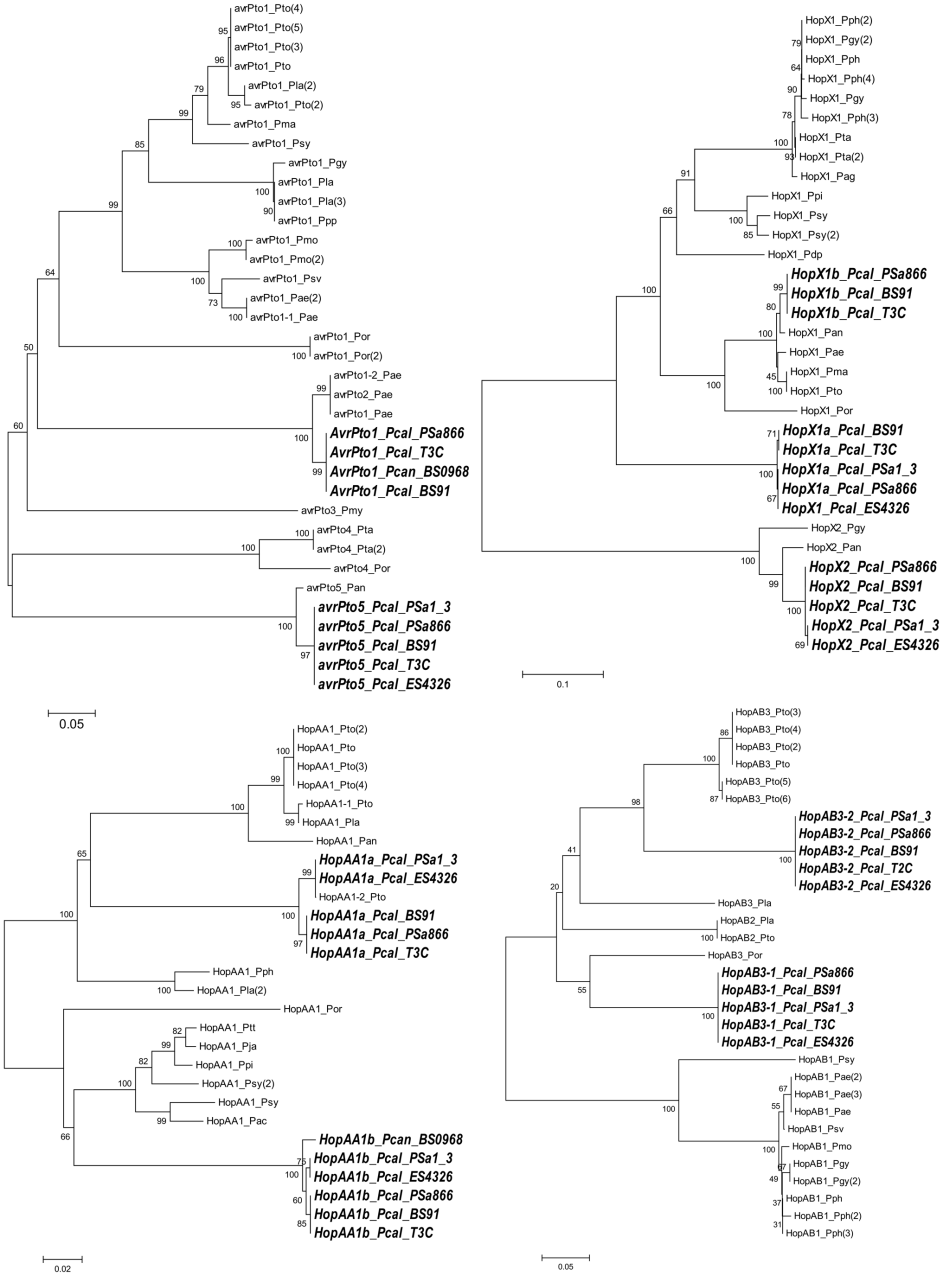
T3SS effector proteins (T3EPs) phylogeny. For the phylogenetic analysis, the amino acid sequences of every effector group were used, as they are presented in the Hop Database website. Additional information for the phylogeny of the rest of *Pcal* T3EPs can be found in figure S4.

We used amino acid sequences of 34 *Pcal* T3EP families, for which there are more than three orthologues in the Hop Database (HDB), for phylogenetic analysis. Cluster comparisons were based on the presence of common and consistent clades (bootstrap values>70). *Pcal* T3EPs were grouped with *P. syringae* T3EPs. Some *Pcal* T3EPs were identical or similar to other known effectors deposited in the HDB. This group included the T3EPs AvrPto1, HopAF1, HopAO1, HopD1, HopO1, HopQ1, HopT1, HopW1-2, HopX1b, HopX2, AvrRpm1, HopZ1, HopG1, HopAW1, HopBF1, HopAQ1, and HopAD1 (Figure 9 and Figure S4). However, HopD1 and HopAQ1 of *Pcal* ES4326 and PSa1_3 clustered separately from their orthologues in the other *Pcal* genomes and *Pcan* BS0968. A second group of *Pcal* T3EPs grouped only weakly with known effectors. This group included orthologs of the effector proteins AvrPto5, HopAZ1, HopBD1, HopR1, HopW1-1, HopE1, HopAM1 (Figure 9 and Figure S4). However, the effector protein HopAM1 of *Pcal* PSa1_3 was phylogenetically divergent from the other *Pcal* and *Pcan* orthologs. A third group of *Pcal* T3EPs were phylogenetically distant from their *P. syringae* orthologues. This group of proteins included the effectors: AvrE1, HopAA1a, HopAA1b, HopAB3-1, HopAB3-2, HopAL1, HopAS1, HopBD2, HopM1, HopI1, HopX1a, HopAU, HopAV1, HopAY1, HopAC1 and HopBG1. It is noteworthy that the effector HopBG1 was found only in *Pcal* pathovars and specifically only in *Pcal* ES4326 and PSa1_3 strains; therefore, due to the lack of HopBG1 orthologues in the HDB we were unable to construct a phylogenetic tree based on this protein.

Some of the strains harbored multiple phylogenetically distinct paralogs of several effectors, which were not likely the products of duplication events. The three *Pcal* strains belonging to Lineage-I (T3C, BS91 and PSa866), as well as *Pcan* BS0968, harbored two AvrPto copies (AvrPto1 and AvrPto5), while the strains of Lineage-II (*Pcal* PSa1_3 and ES4326) carried only one copy (AvrPto5). On the other hand, Lineage-I strains carried three HopX xenologs loci (HopX1a, HopX1b and HopX2), while the Lineage-II strains, *Pcal* PSa1_3 and ES4326, as well as the *Pcan* BS0968, harbored only two (HopX1a and HopX2) (Figure 9). The *Pcal* HopX1b orthologues resembled HopX1 from *Pst* DC3000 that has been reported to enhance RNA-mediated gene silencing (RNAi) in *Nicotiana benthamiana* independently of plant R-gene recognition [52]. Therefore, it will be interesting to examine if the newly identified *Pcal* HopX proteins have a similar activity. All strains examined harbored two copies of the T3EP HopAA (HopAA1a and HopAA1b), HopAB (HopAB3-1 and HopAB3-2) and HopW (HopW1-1 and HopW1-2). Lastly, HopBD1 was present only in Lineage-II strains (*Pcal* PSa1_3 and ES4326), each carrying two divergent copies (HopBD1 and HopBD2; Figures 8A and Figure S4).

### Type VI Secretion System Gene Clusters in the *P. cannabina* pv. *alisalensis* Genomes

**Figure 10:**
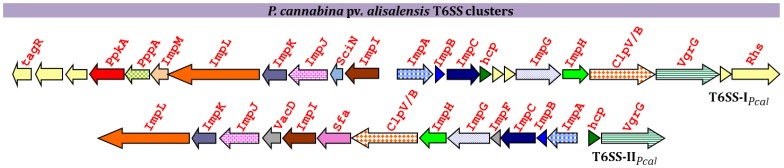
Schematic representation of the gene *cluster* coding for the type VI secretion systems (T6SSs) of *Pseudomonas cannabina* pv. *alisalensis*.

Our analysis of *Pcal* genomes revealed two genomic clusters coding for putative T6SSs (T6SS-I and T6SS-II). The T6SS-I locus consisted of a set of 16 conserved T6SS core genes (Figure 10). This locus is syntenic and phylogenetically closely related to the *P. aeruginosa* (*Paer*) T6SS-I [53] (formally HSI-I; *Pseudomonas* spp. T6SS group 3, according to Barret et al., [54]), with the exception of a replacement of the *P. aeruginosa pppB* gene with *impM* in the *Pcal* genome and an insertion of two putative ORFs apparently unrelated to type VI secretion that interrupt the synteny of the *hcp* and *impG* ORFs. The HSI-I type of T6SS is also present in several other *Pseudomonas* species, including the plant root-associated bacterium *Pseudomonas brassicacearum* subsp. *brassicacearum* NFM421.

In addition to structural core proteins that serve as landmarks of T6SS loci, a serine-threonine protein kinase gene with homology to *ppkA* (Stk1), as well as its cognate phosphoprotein phosphatase *pppA* (Stp1), were also found within the *Pcal* T6SS-I locus (Figure 10). We also searched for the presence of the *tse* (*tse1, tse2* and *tse3*) toxin genes. Tse2 (PA2702) is a toxic protein, first described in *P. aeruginosa* strain PAO1, that affects growth of both prokaryotic and eukaryotic cells when expressed intra-cellularly and is a proposed substrate of the *P. aeruginosa* T6SS-I [55]. *P. aeruginosa* also codes for an immunity protein, Tsi2 (PA2703) that prevents Tse2-dependent cell death [55]. We did not identify any orthologs of either *tse1, tse3*, or the *tse2*/*tsi2* regulon in any of the *Pcal* genomes examined. Since these genes were missing from all five draft genomes this is probably not due to poor genome coverage but rather was likely a reflection of differences in biology between *P. aeruginosa* and *Pcal*.

The second T6SS cluster (T6SS-II) in the *Pcal* genomes was highly similar to the T6SS-II cluster of *Pst* DC3000 (*Pseudomonas* spp. T6SS group 1.1) [23], [24], [54], except that the *Pcal* cluster lacked the *pppA* and *ppkA* regulatory genes. Again, the absence of these two genes from all five draft genome sequences suggested that the absence of these genes was not an artifact of poor genome coverage.

### Phylogenetic Analysis of *P. cannabina* pv. *alisalensis* T6SS Proteins

Two phylogenetic trees were constructed using the T6SS core proteins ImpL (Figure S5) and ClpV/B (Figure S6). Various *Pseudomonas* species exhibiting different phylogenetic relatedness to *Pcal* were included. Both phylogenetic trees showed three deep branches. The first deep branch in the ImpL tree (marked as *Paer* and *Pcal* group), contained only *Pcal* T6SS-I along with T6SS-I of *P. aeruginosa*, which were phylogenetically separated from all other *P. syringae* and *P. aeruginosa* T6SSs. The next two branches were as described previously [23], [24]; the first (*Psph* group) included the *P. syringae* T6SSs-I, while the second branch (*Pst* group) comprised the *Pcal* T6SS-II, the *P. syringae* T6SS-II and T6SS-III along with the sole T6SS of *Pseudomonas fluorescens* Pf0-1, *P. aeruginosa* T6SS-II and *P. entomophila* T6SS-II. The *P. aeruginosa* T6SS-III was present as a separate group in the ImpL phylogeny. The ClpV/B tree had three branches (Figure S6). However, while the *Pcal* T6SS-I grouped again with *Paer* T6SS-I (*Pcal* and *Paer* group), the *P. syringae* T6SS-I branched separately as previously reported [23], [24].

Phylogenetic analysis of the T6SS loci provides some insight into the potential function of these T6SS-I: it tightly links the *Pcal* T6SS-I clusters with the *P. aeruginosa* T6SS-I, (HSI-1) which is proposed to be essential for inter-bacterial interactions [55], [56], suggesting a similar role for the *Pcal* T6SS-I.

However, unlike *P. syringae*, *P. aeruginosa* and other *Pseudomonas* species the *Pcal* strains did not harbor multiple copies of *vgrG* and *hcp* genes [23], [53].

### Bacterial Toxin Genes and Gene Clusters

Our analyses revealed the presence of genes and gene clusters coding for: a) coronatine biosynthesis, with high levels of similarity to the *Pst* DC3000 coronamic acid synthetases: 100% identity with the CmaD (PSPTO_4707), 99% identity with CmaE (PSPTO_4708), 93% identity with the CmaA (PSPTO_4709), 100% identity with the CmaB (PSPTO_4710), 100% identity with the CmaC (PSPTO_4711) and finally 99% identity with CmaT (PSPTO_4712), b) An ethylene-forming enzyme (*efe*). Although the IAA-lysine synthetase (*iaaL*) was present in all five *Pcal* strains, with 96% identity with the IaaL ortholog of the *Pst* DC3000 (PSPTO_0371), we did not identify orthologs of the *iaaM*/*iaaH* genes. Furthermore, all five *Pcal* strains possessed a gene coding for tabtoxin resistance protein (TTR) with identity 92% to the tabtoxin resistance protein of *Pseudomonas syringae* pv. *tabaci* strain ATCC 11528 (PsyrptA_8578), an enzyme involved in self-protection found in tabtoxin-producing pathogens. Interestingly, there were several remnants of what appear to be members of the tabtoxin biosynthesis gene cluster in the *Pcal* genomes.

### Conclusion

Comparison of five *P. cannabina* pv. *alisalensis* genomes with each other and with several *P. syringae* genomes revealed that virulence genes have been exchanged between these pathogens during their evolution and that virulence mechanisms and regulation of virulence gene repertoires between *P. syringae* and *P. cannabina* largely overlap.

Within *Pcal*, differences between strains with regard to T3EP repertoires reflected the existence of two genetic lineages that were previously identified [3]. Interestingly, although strains belonging to these two lineages were isolated from different crops no host range differences could be identified experimentally suggesting that the two lineages may have separated by factors other than host range evolution. However, experimental manipulation of T3EP repertoires may still reveal differential roles of effectors in virulence on different hosts. Likewise, analysis of *Pcal* genomes showed two genomic clusters coding for T6SSs (T6SS-I and T6SS-II), the first of which is syntenic and related to the *P. aeruginosa* (*Paer*) T6SS-I. However, the possible contribution of the T6SS in pathogenicity of *Pcal* species remains to be studied.

*Pcal* is a particularly interesting pathogen because it can cause disease on both monocots and dicots. Now that the virulence gene repertoires of *Pcal* have been predicted, roles for these genes in pathogenicity can be investigated in a variety of plant systems. Such studies will not only provide mechanistic insights into molecular interactions underlying virulence, but also provide a framework for understanding selection pressures that shape evolutionary dynamics for bacterial phytopathogens across divergent plant hosts.

## Materials and Methods

### Bacterial Strains

To evaluate the core genome and strain specific characters for *Pcal*, we sequenced four strains selected in order to differ in the genera of host plant and geographic origin: the pathotype strain *Pcal* BS91 (CFBP 6866) which was isolated in USA from *Brassica rapa* subsp. *rapa* [3]; the *Pcal* strain T3C (CFBP 7684), which was isolated from the Turnip cv. ‘topper’ in Lexington, South Carolina [39]; the strain *Pcal* PSa1_3 (CFBP 7682) that was isolated in Greece from *Eruca sativa* [10]; the strain *Pcal* PSa866 (CFBP 7683) a re-isolation from artificially infected tomato plants with a *Pcal* strain of unknown origin. Additionally, we used the genomic sequence available for *Pcal* ES4326 for comparisons with the other *Pcal* strains [37]. All strains were isolated from symptomatic plants revealing the typical symptoms and were used for successful artificial inoculations on various plant species (Figures 1 and S1).

### Bacterial Cultures and Genomic DNA Preparation

The strains examined were grown at 26–28°C in King’s medium B broth for 24 h. From these cultures, cells were washed with sterile 10 mM MgCl_2_, and a cell suspension was prepared, which was adjusted to an OD_600_ of 0.4. Cultures were stored in aliquots of 500 µL KB in 2 mL cryo-tubes at −80°C. For DNA extraction, bacterial cells from over-night cultures were spun down, and the pellets kept on ice before further use. Total bacterial DNA isolation was carried out using the DNeasy Blood & Tissue Kit from QIAGEN (UK) according to the manufacturer’s instructions.

### Library Preparation and Sequencing

Library preparation for *Pcal* PSa866 and *Pcal* PSa1_3 sequencing was performed with the Ion Fragment Library Kit (Life Technologies, Darmstadt, Germany) according to the protocol (part no. 4467320 rev. A, 04/2011) with minor modifications. Size selection was done with E-GelH Size Select 2% Agarose (Invitrogen, UK) for strains *Pcal* PSa1_3 and *Pcal* PSa866. Template preparation was carried out with the Ion Xpress TM Template Kit (Life Technologies) according to the Ion Xpress TM Template Kit User Guide (part no. 4467389 Rev. B, 05/2011). Emulsified Ion Sphere TM particles were collected by centrifugation (2200 g for 8 min) in a SOLiDH emulsion collection tray (Life Technologies). After centrifugation a clear oil phase developed above a white solid pellet. The oil layer was decanted and pelleted Ion Spheres were re-suspended with 700 µl of breaking solution followed by two washes of the emulsion collection tray with breaking solution. In a departure from the User Guide, all three fractions were pooled in the same 2 ml reaction tube. Washing of the recovered Ion Sphere particles was performed as described in the protocol. The Ion Sequencing Kit (Life Technologies) was used with the Personal Genome Machine TM (PGM TM) sequencer as described in the Ion Sequencing Kit User Guide (part no. 4467391 rev. B, 04/2011). Enriched ISPs were prepared for sequencing as described in the protocol and deposited on the chip in three consecutive loading cycles. Each cycle was composed of the following steps: (i) adjust sample volume to 19 µl with annealing buffer, (ii) 10 sec sonication followed by a quick spin, (iii) re-suspension by pipetting and loading of 6 µl of the sample to the chip, and (iv) 3 min centrifugation using the custom centrifuge adapter/rotor.

Genomic DNA of strains *Pcal* BS91 and *Pcal* T3C was extracted using the Gentra Puregene Yeast/Bacteria kit (Qiagen) following manufacturer’s instructions. Illumina paired-end sequencing was performed as previously described [57].

### Whole-genome Assembly and Alignment of Illumina and Ion Torrent Genomes

For *Pcal* PSa866 and *Pcal* PSa1_3 paired reads of about 100 nts were assembled into contigs using the *de novo* assembly, as well as using the reference assembling option of the CLC genomic workbench (CLC-bio, Aarhus, Denmark) using as reference the *Pcal* ES4326 genome [37]. Gene calling was performed by genomic annotation tools of JGI/IMG-ER (https://img.jgi.doe.gov/cgi-bin/er/main.cgi). The genome sequence analysis was performed with the aid of the software package MAUVE [40], specialized for construction of multiple genome alignments in the presence of large-scale evolutionary events such as rearrangements and inversions. Additional assessment of the effector genes repertoire was performed based upon the methods of Baltrus [37].

For *Pcal* BS91 and T3C paired-end reads were assembled de novo using Velvet 0.7.55 [58] as previously described [57] with scaffolding turned on.

### Phylogenetic Analyses

Phylogenetic analysis of specific genes was carried out using the sequences obtained from all sequenced strains plus corresponding sequences retrieved from the GenBank. Sequence alignments were carried out using the program CLUSTALW [59]. The phylogenetic trees were established using the Neighbour-Joining method [60]. The percentage of replicate trees in which the associated strains clustered together in the bootstrap test for 1500 replicates [61] was estimated and is shown next to the tree nodes. The trees were drawn to scale, with branch lengths in the same units as those of the evolutionary distances used to infer the phylogenetic trees. The evolutionary distances were computed using the Maximum Composite Likelihood method [62] and are in the units of the number of base substitutions per site. All positions containing gaps and missing data were eliminated from the dataset (complete deletion option). Phylogenetic analyses of specific genes were conducted in MEGA5 [63].

The whole genome phylogenetic analysis was conducted using all-against-all Blastp [64] comparisons of protein sequences of 41 genomes (http://pacu.facom.ufms.br/Pcal/genomes). Sequences were clustered in 13.607 families using OrthoMCL [65]. From this total, 710 families were selected that contained exactly one protein per genome (except for the outgroup genomes of the *P. fluorescens* strains, which contained a maximum of one protein per family). Each of the 710 families was then aligned using MUSCLE [66]. Non-informative columns of each alignment were removed using Gblocks [67] and all alignments were concatenated, resulting in 210,004 positions. Finally, RAxML [68] with model PROTGAMMAWAGF was used on this alignment to build the final species tree, shown in Figure 2.

### Pathogenicity Tests

In preliminary assays all isolates were screened for their ability to induce hypersensitive reaction on tobacco leaves and to cause typical symptoms on their natural hosts by previously described methods [3], [10]. All plants used for inoculations, were grown in separate 20 cm diameter pots in a 3∶1:1 compost, peat and perlite mix. Plants were watered with surface drip irrigation. Fertilizer 20-20-20 (N-P-K) was applied weekly by watering. Inoculations were carried out on known host plants. Ten plants of each host were inoculated with the examined strains (Figures 1 and S1). For foliar inoculations on the host plants, a suspension of each isolate was sprayed onto leaves until run off with a hand sprayer. The bacterial inocula were prepared from 24 hrs old King’s medium B plate cultures, suspended in sterile distilled water and adjusted to approximately 10^6^ CFU*·*mL^−1^ based on turbidity measurement at 600 nm and by dilution plate counts. Control plants were sprayed with sterile distilled water. Inoculations of tomato plants were performed by first slightly wounding the plant leaves and stems with a soft plastic brush immediately prior to spray inoculation as described above. Controls were similarly treated. All inoculated plants were held in a greenhouse (10–30°C) under intermittent mist (10 sec each hour) and symptoms were evaluated for two weeks after inoculation. Cross-inoculation tests were made on several plant species (Figures 1 and S1). Bacterial colonies were re-isolated from infected plants and the re-isolates had the same LOPAT profile as the original isolates of *Pcal*. Isolates from control plants did not reveal any pathogenic bacteria, and published host ranges were confirmed [3], [10], [18], [39], [69].

## Supporting Information

Figure S1 Compatible reactions to artificial inoculations of *Pseudomonas cannabina* pv. *alisalensis* (*Pcal*) on various plant species. Artificial inoculations were performed using the sequenced *Pcal* strain PSa1_3 on: *Brassica oleracea*, *Eruca sativa*, and *Brassica napus*. Information for additional artificial inoculations on other plant species can be found in Figure 2.
(PDF)

Figure S2 Pairwise alignment between the compete genome of *P. s.* pv. *tomato* DC3000 and the draft genome of *Pcal* ES4326 (previously known as *P. s.* pv. *maculicola* ES4326) (**A**) and the draft genomes of *Pcal* PSa1_3 and *Pcal* PSa866 (**B**) using the MAUVE software. Colored blocks outline genome sequence that aligned to part of another genome, and is presumably homologous and internally free from genomic rearrangement (Locally Colinear Blocks or LCBs). Areas that are completely white were not aligned and probably contained sequence elements specific to a particular genome. Blocks below the center line indicate regions that aligned in the reverse complement (inverse) orientation.
(PDF)

Figure S3 T3SS core component HrpZ and HrcC phylogenetic analysis. For the phylogenetic analysis the amino acids, as well as the nucleotide sequences were used.
(PDF)

Figure S4 T3SS effector proteins (T3EPs) phylogeny. For the phylogenetic analysis, the amino acid sequences of the *Pcal* effector as well as of other effectors, as they are presented in the Hop Database website, were used. Additional information for the phylogeny of the rest of *Pcal* T3EPs can be found in Figure S8.
(PDF)

Figure S5 Phylogenetic analysis based on the protein sequences of the T6SS component, ImpL. For the phylogenetic analysis the amino acid sequences were used. Information for additional phylogenetic analysis of various T6SS core components can be found in figure S6.
(PDF)

Figure S6 Phylogenetic analysis based on the T6SS protein sequences of the T6SS ATPase, ClpV/B. For the phylogenetic analysis the amino acid sequences were used. Information for additional phylogenetic analysis of various T6SS core components can be found in figure S5.
(PDF)

Table S1 Single nucleotide polymorphisms between *Pcal* PSa1_3, PSa866 and T3C *hrp*/*hrc* clusters.
(DOCX)

Table S2 Plasmids present in *Pcal* strains.
(DOCX)

Table S3 Genomes used for the phylogenetic tree shown in Figure 2 and for the comparison of protein repertoires that can be queried at http://pacu.facom.ufms.br/Pcal/.
(XLSX)
