# Supplementary figures and images for: Comparative Genomics of Multiple Strains of Pseudomonas cannabina pv. alisalensis, a Potential Model Pathogen of Both Monocots and Dicots
Source: PLoS One. 2013 Mar 28;8(3):e59366. doi: 10.1371/journal.pone.0059366 (PMC3610874; doi:10.1371/journal.pone.0059366)

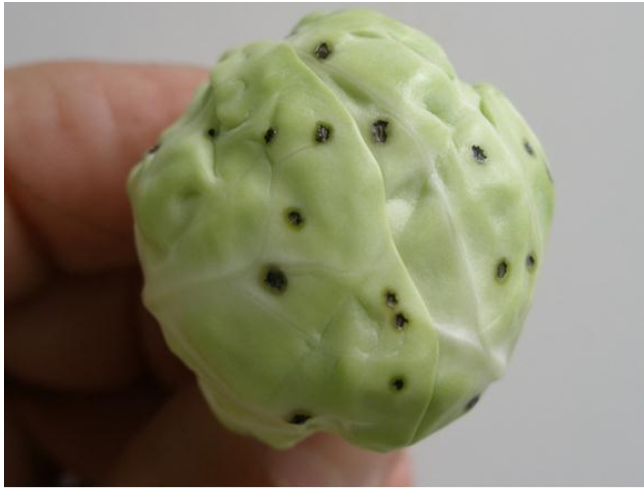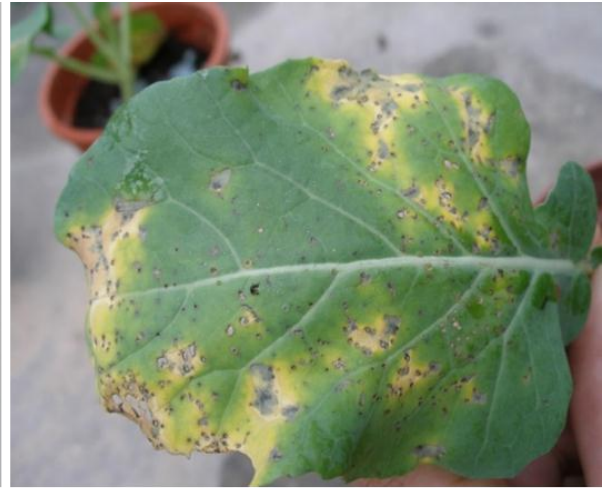

***Brassica oleracea***

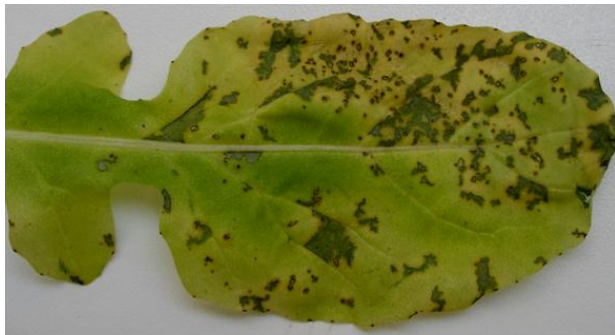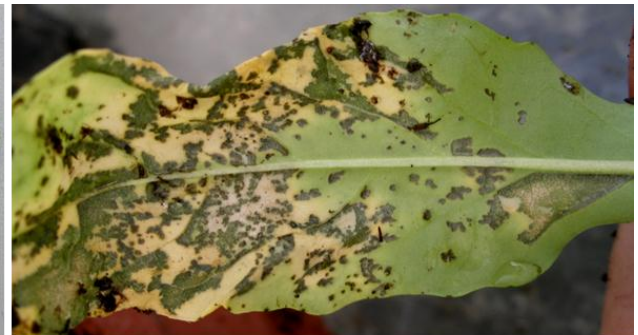

***Eruca sativa***

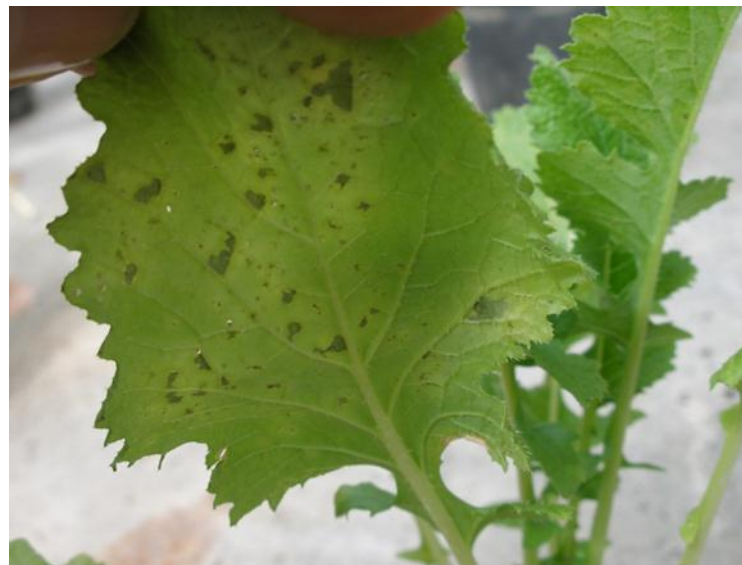

***Brassica napus***

Figure S1

Supplement: Figure S1 — Compatible reactions to artificial inoculations of Pseudomonas cannabina pv. alisalensis (Pcal) on various plant species. Artificial inoculations were performed using the sequenced Pcal strain PSa1_3 on: Brassica oleracea, Eruca sativa, and Brassica napus. Information for additional artificial inoculations on other plant species can be found in Figure 2. (PDF) [file pone.0059366.s001.pdf]

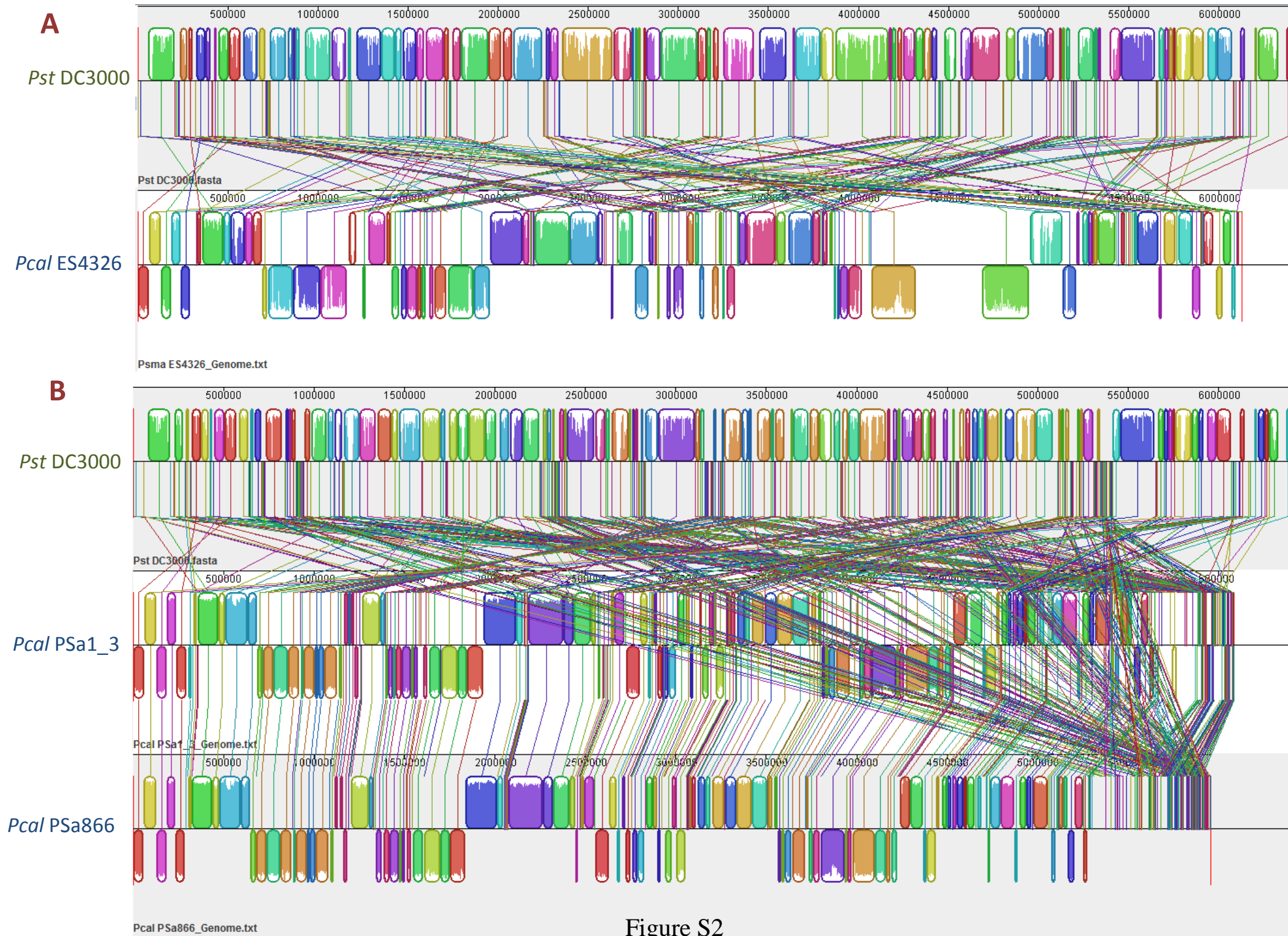

Figure S2

Supplement: Figure S2 — Pairwise alignment between the compete genome of P. s. pv. tomato DC3000 and the draft genome of Pcal ES4326 (previously known as P. s. pv. maculicola ES4326) (A) and the draft genomes of Pcal PSa1_3 and Pcal PSa866 (B) using the MAUVE software. Colored blocks outline genome sequence that aligned to part of another genome, and is presumably homologous and internally free from genomic rearrangement (Locally Colinear Blocks or LCBs). Areas that are completely white were not aligned and probably contained sequence elements specific to a particular genome. Blocks below the center line indicate regions that aligned in the reverse complement (inverse) orientation. (PDF) [file pone.0059366.s002.pdf]

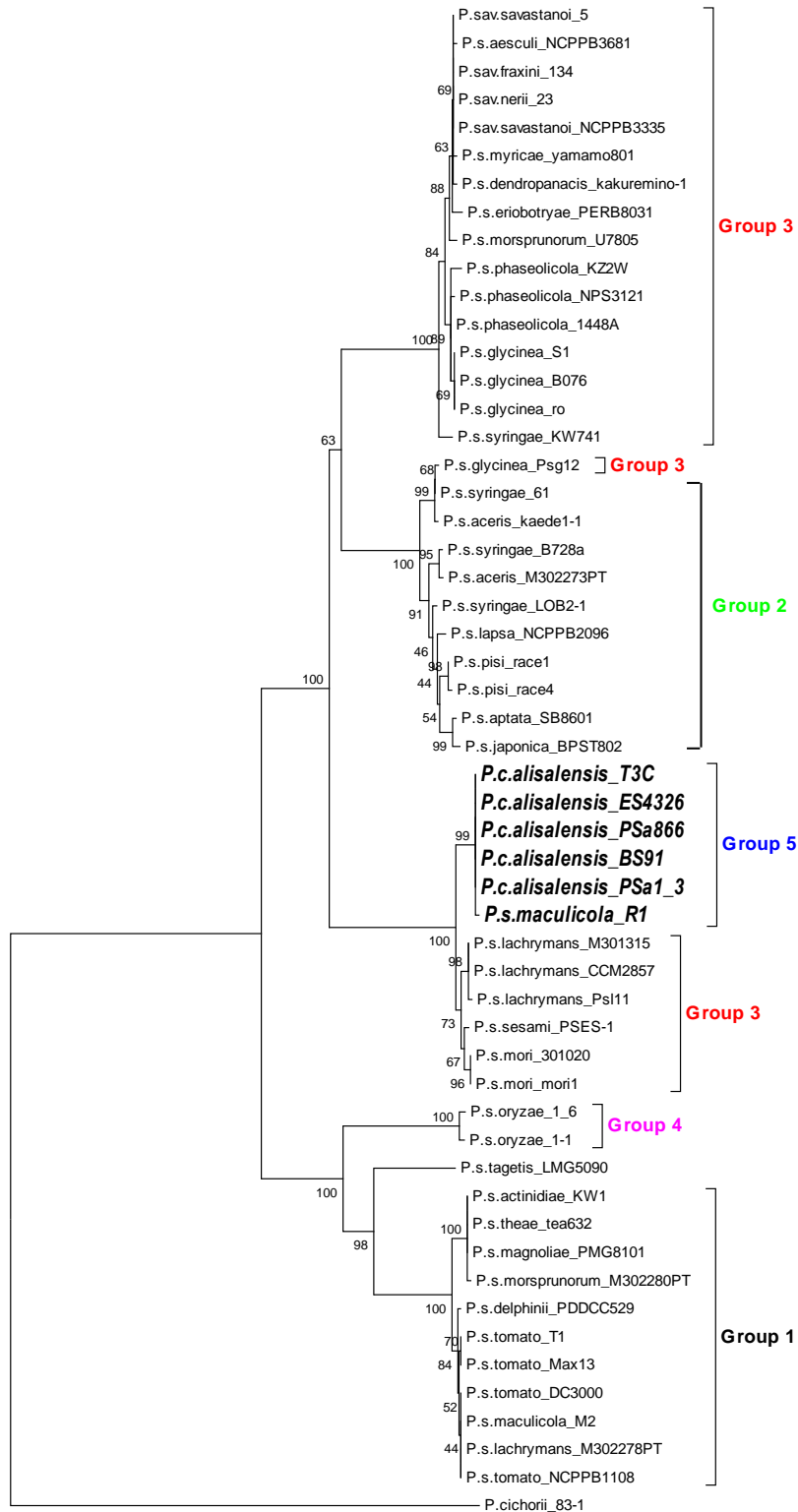

HrpZ : Amino-acid based phylogenetic tree

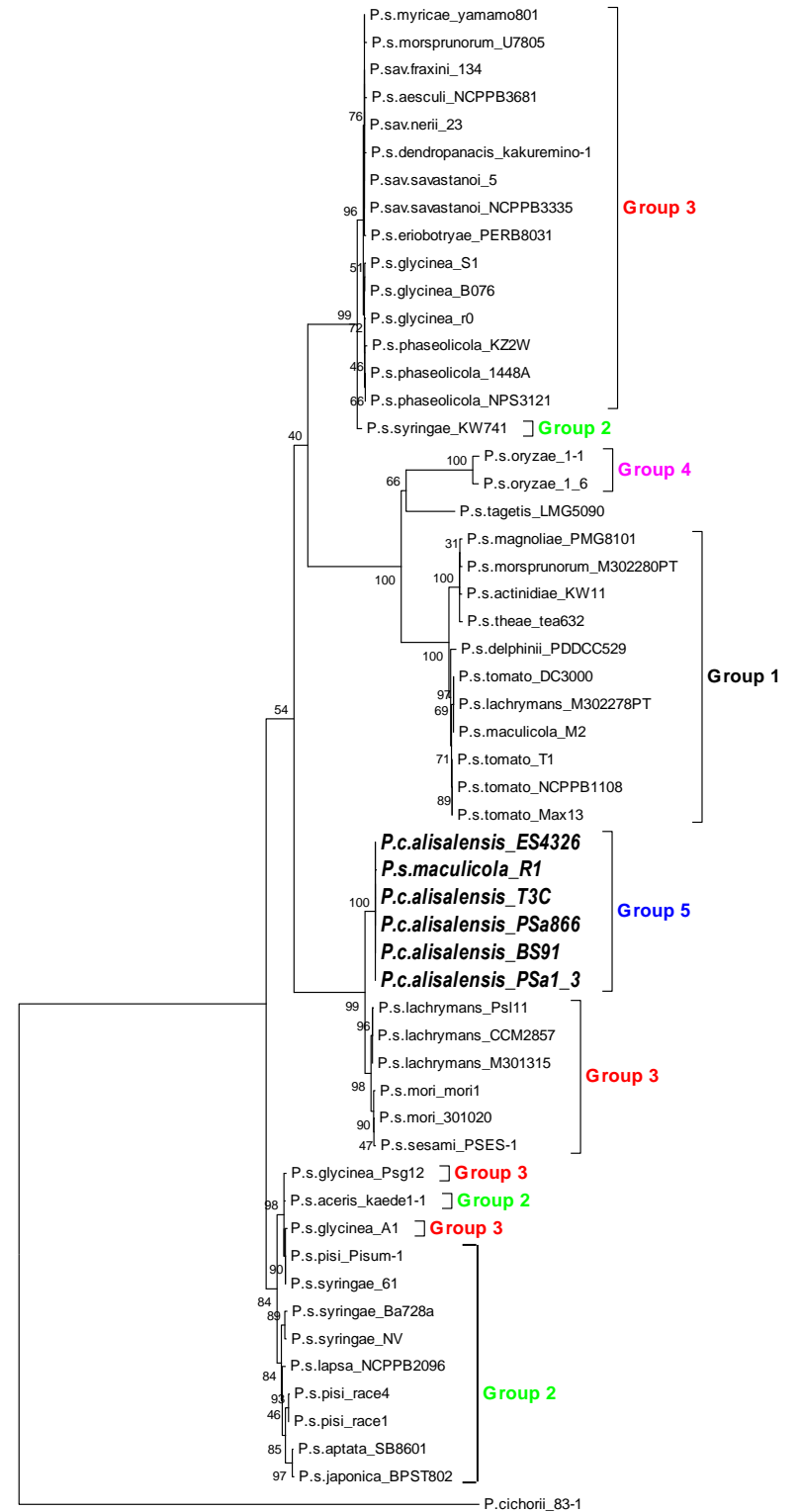

hrpZ : Nucleotide based phylogenetic tree

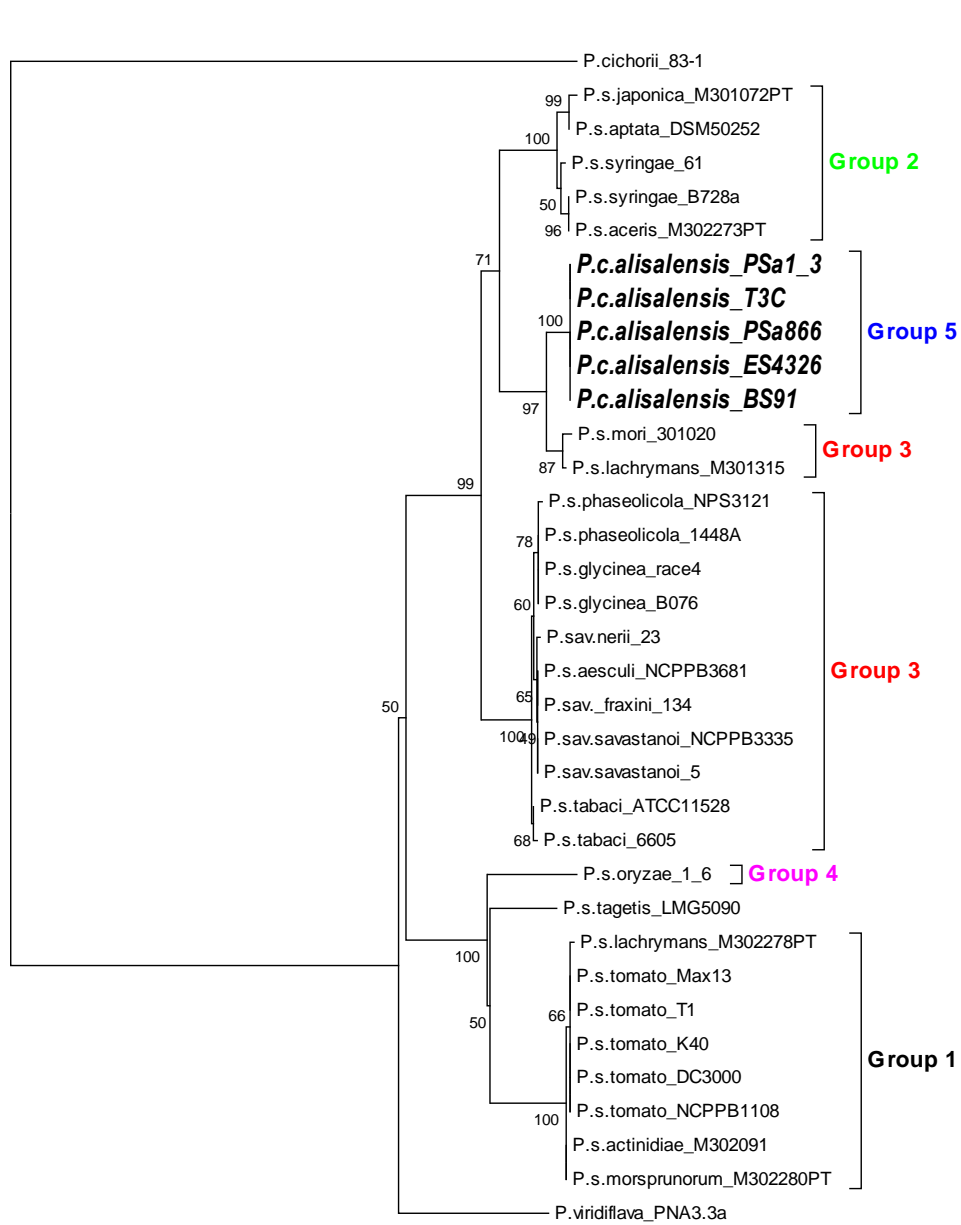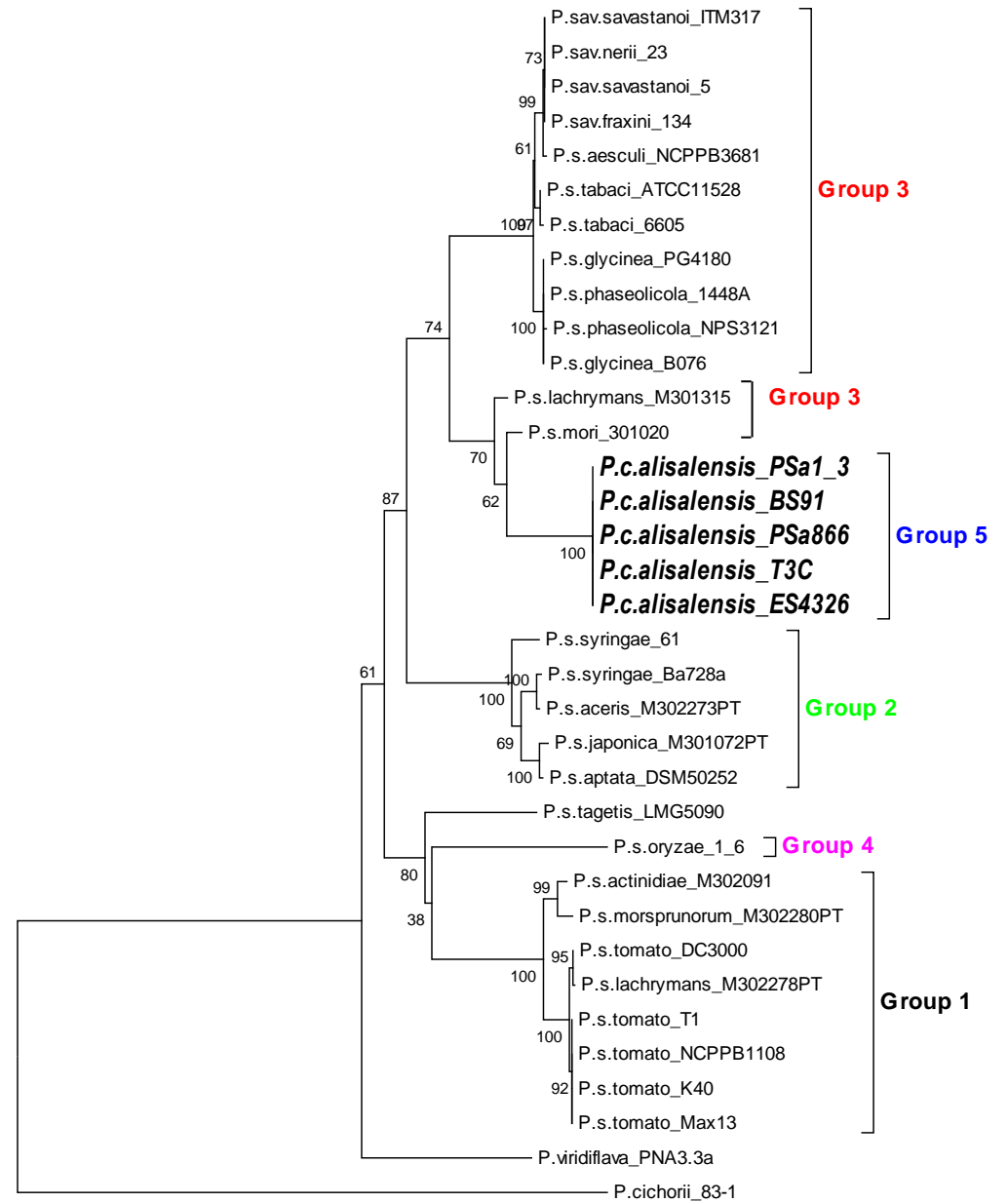

Figure S3

Supplement: Figure S3 — T3SS core component HrpZ and HrcC phylogenetic analysis. For the phylogenetic analysis the amino acids, as well as the nucleotide sequences were used. (PDF) [file pone.0059366.s003.pdf]

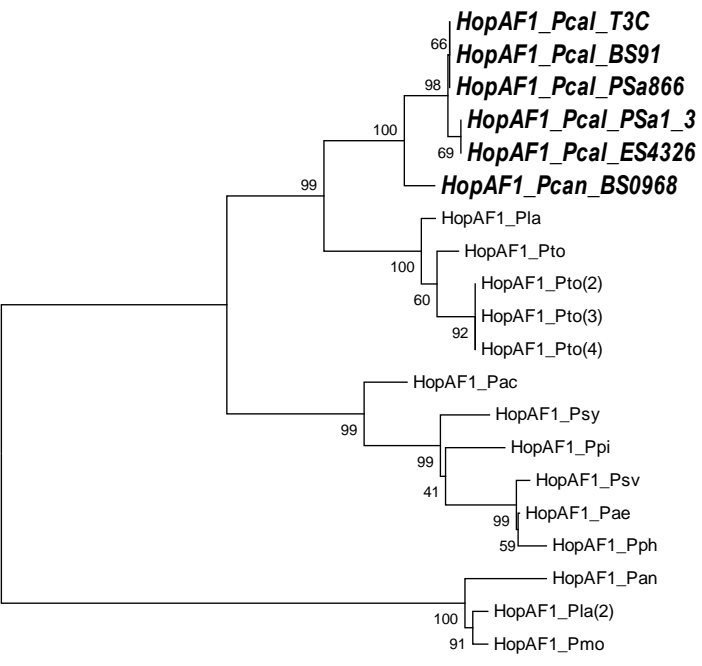

0.02

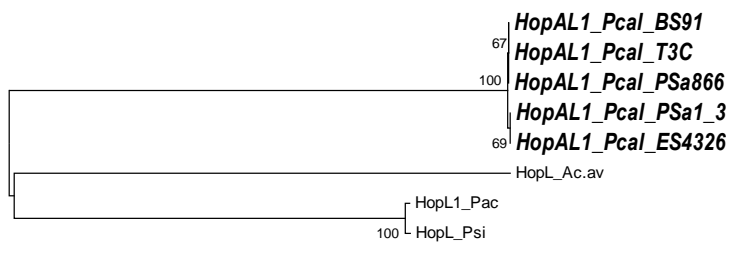

0.1

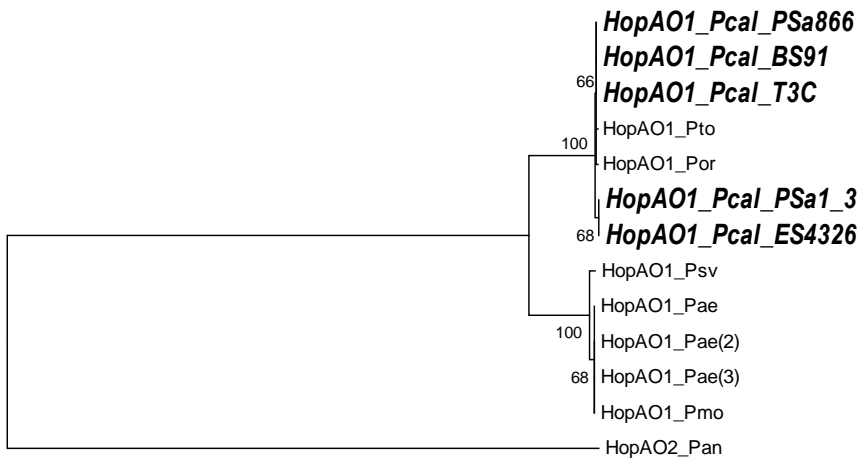

0.05

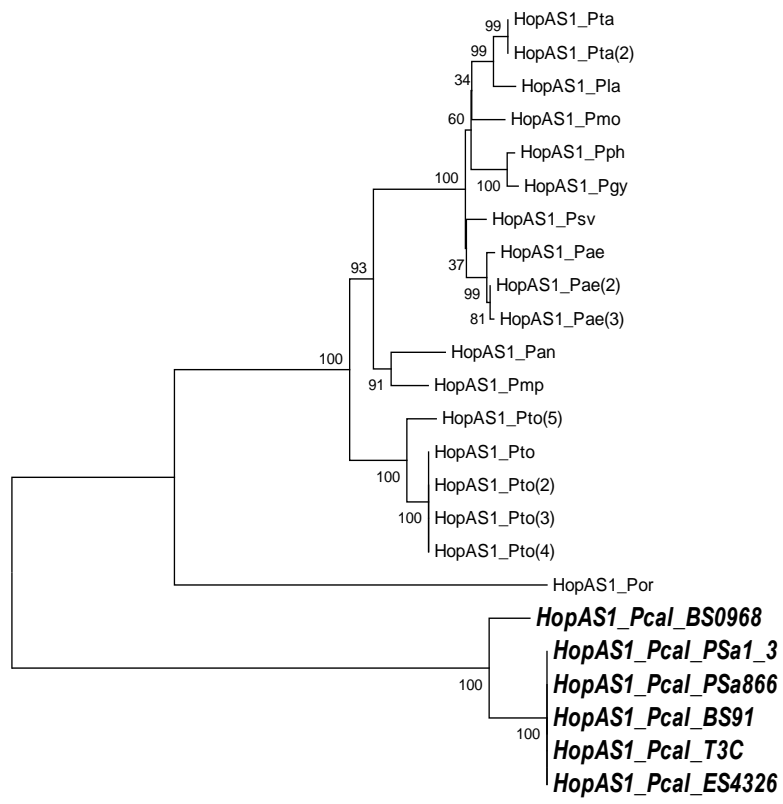

0.02

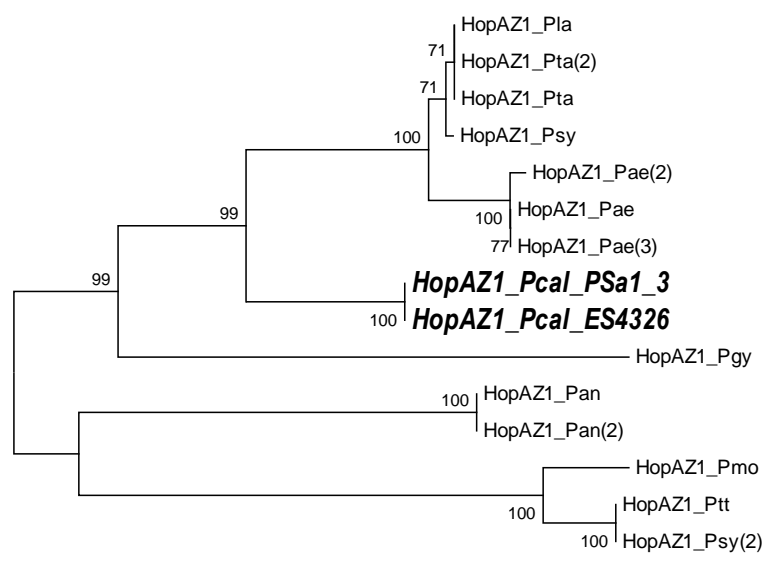

0.05

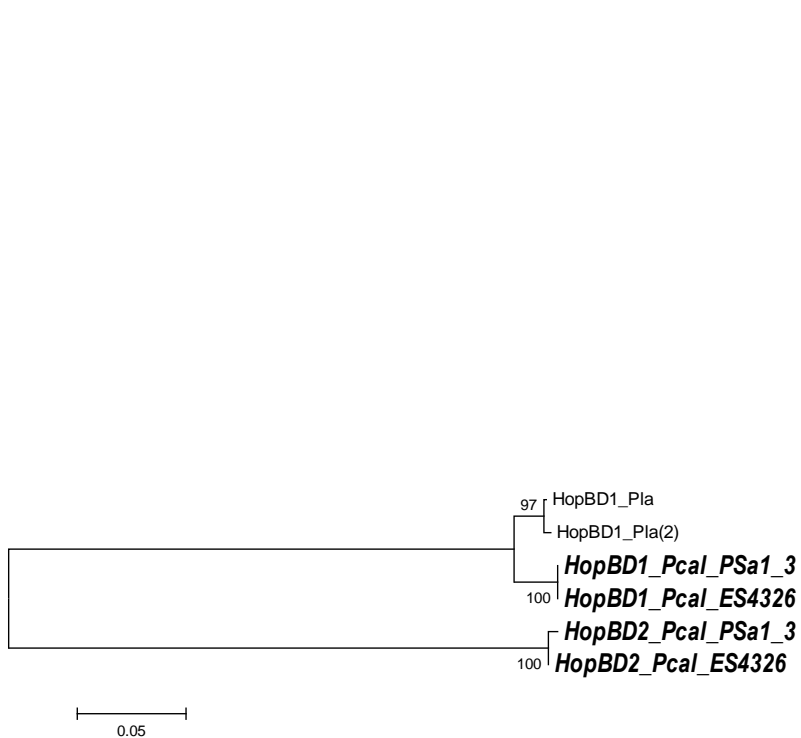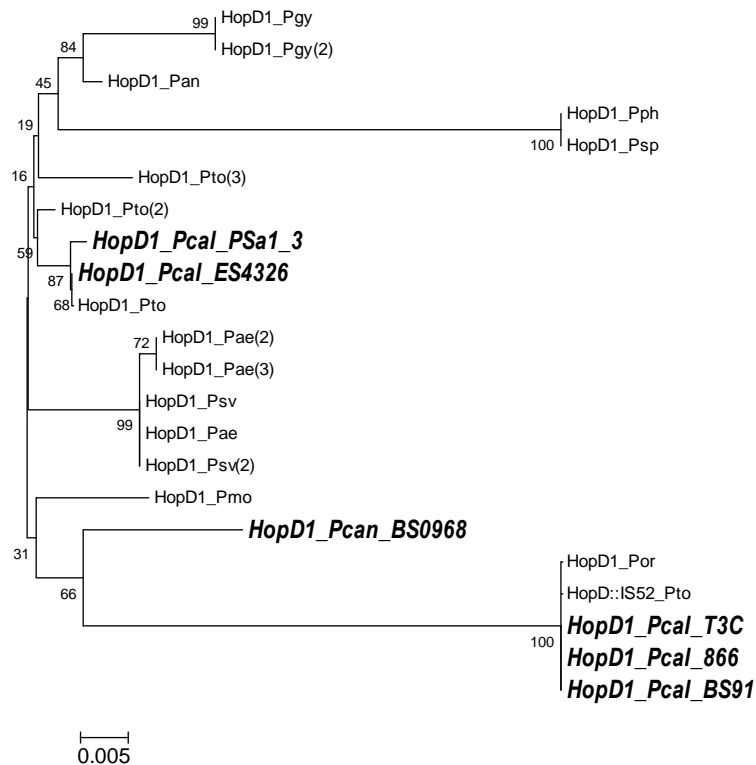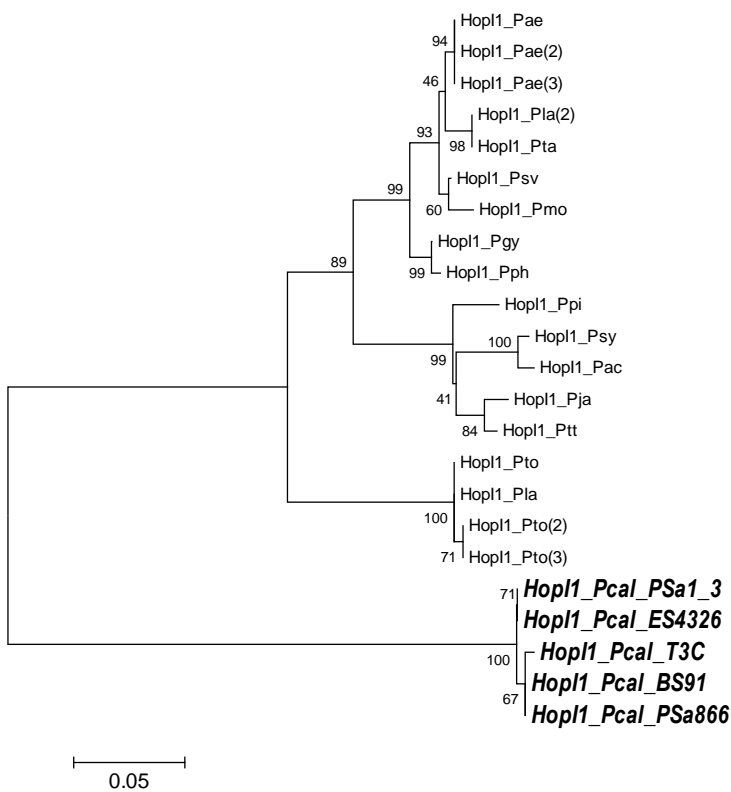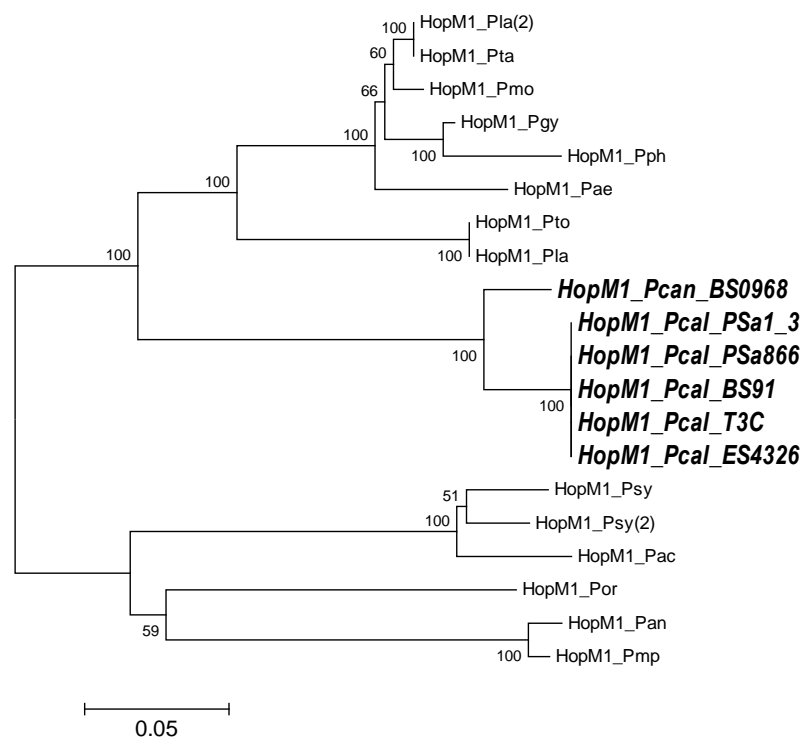

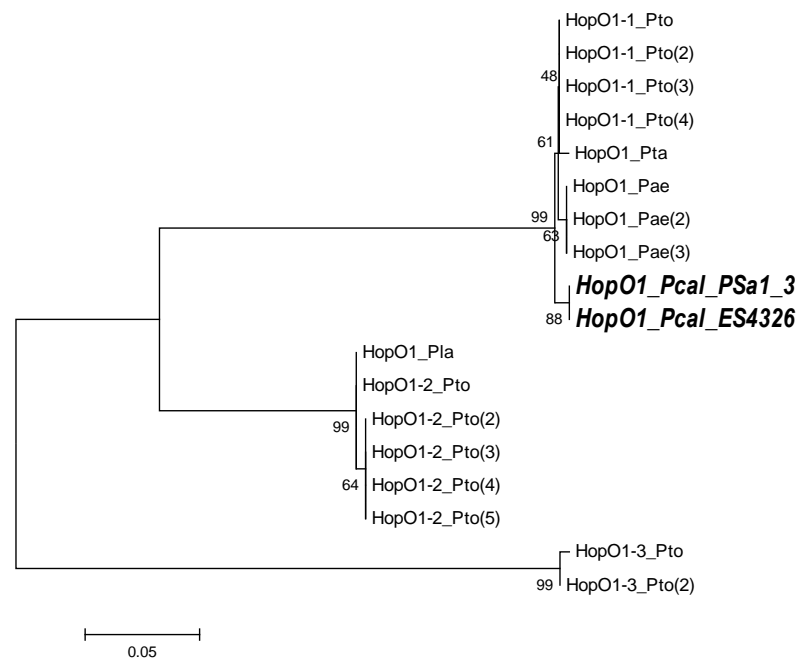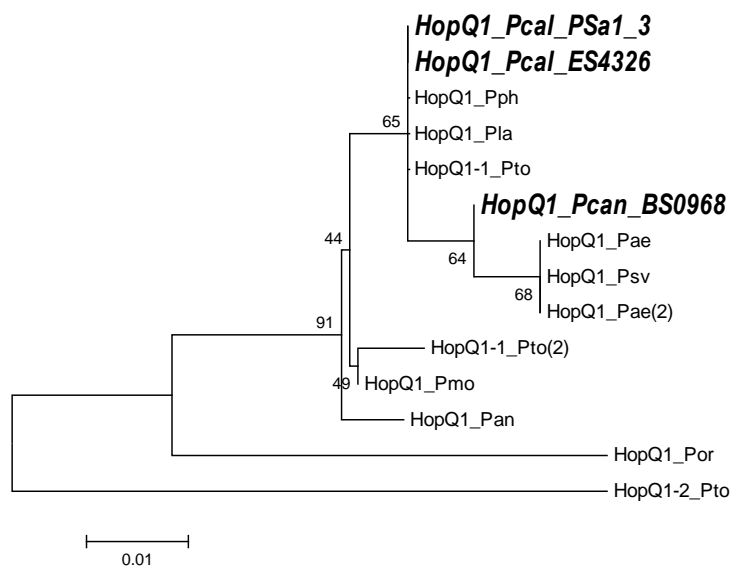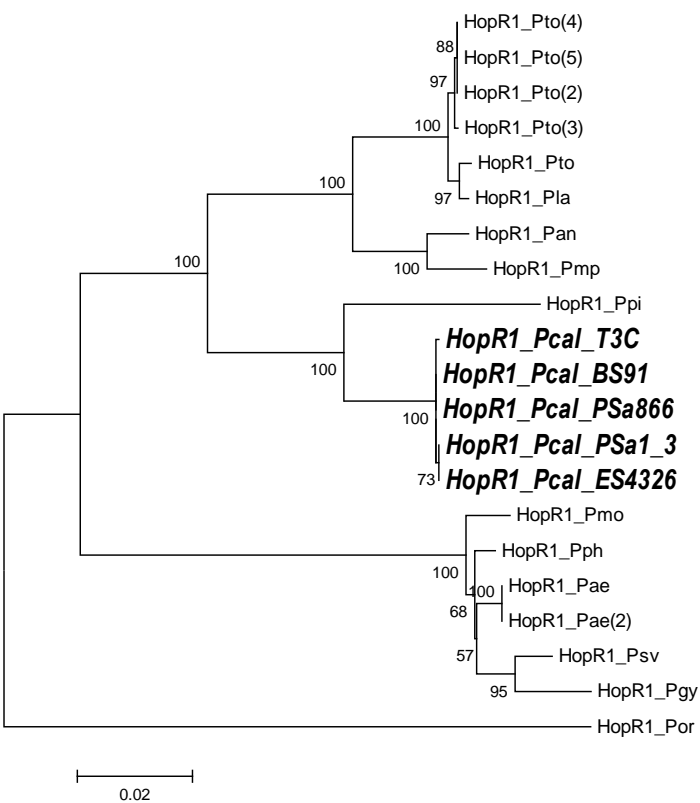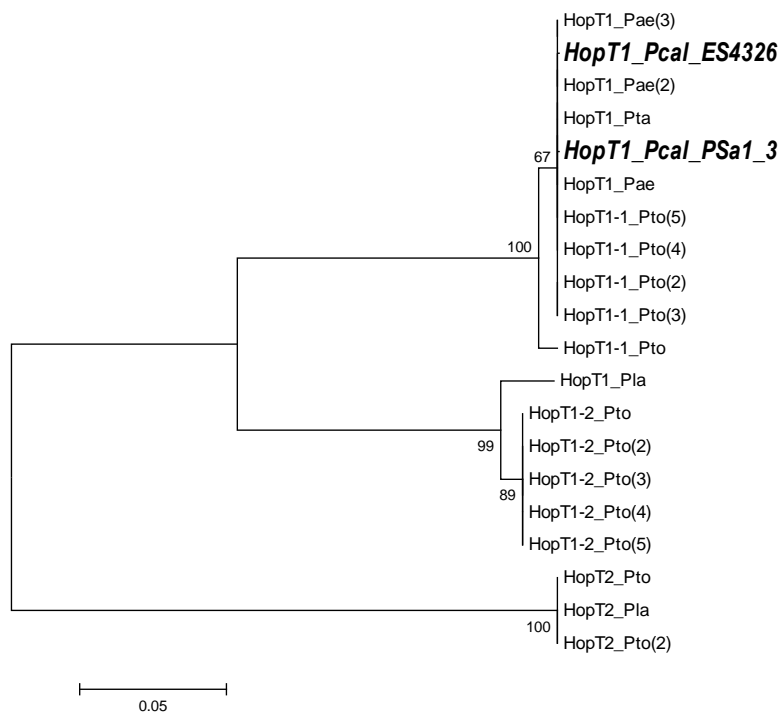

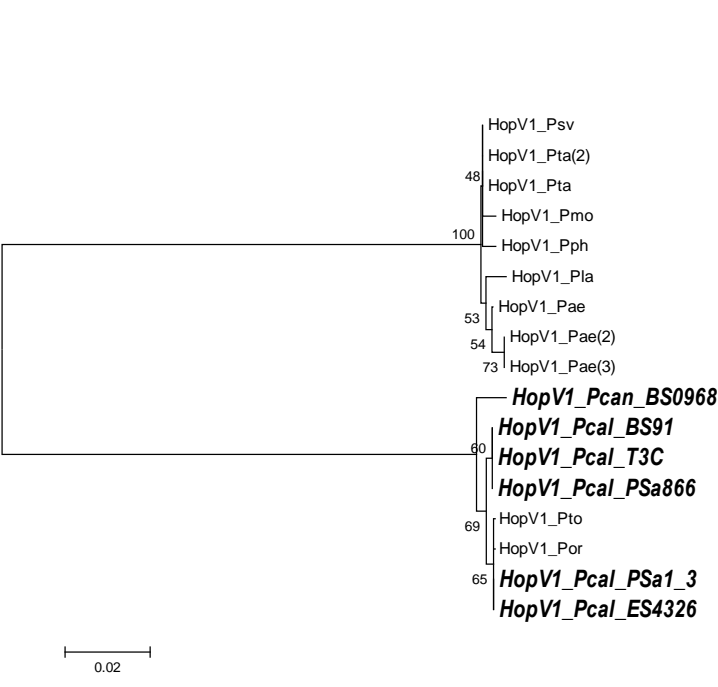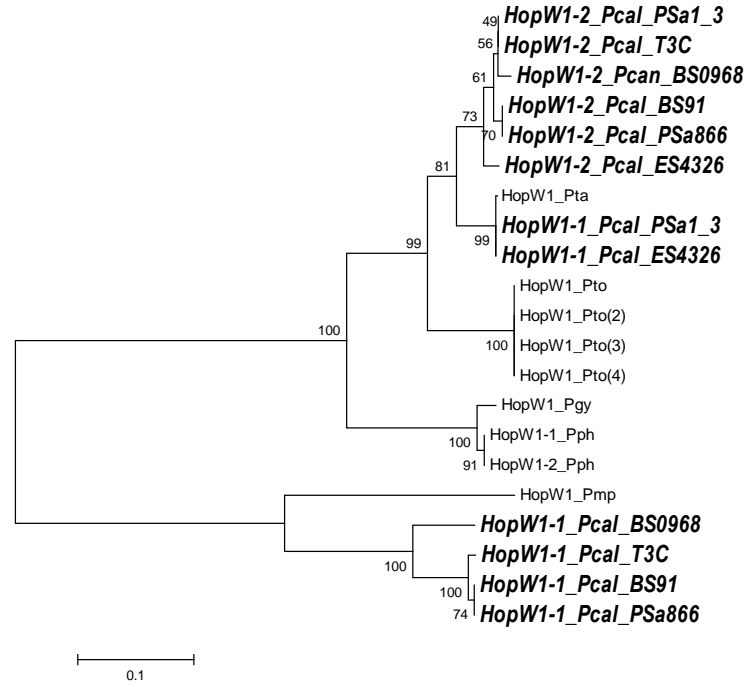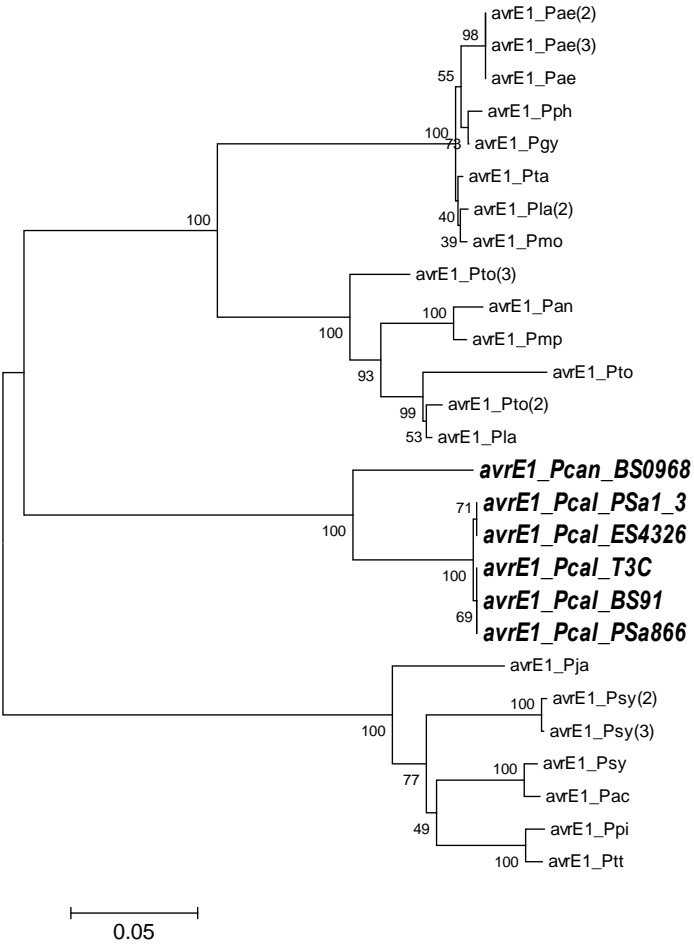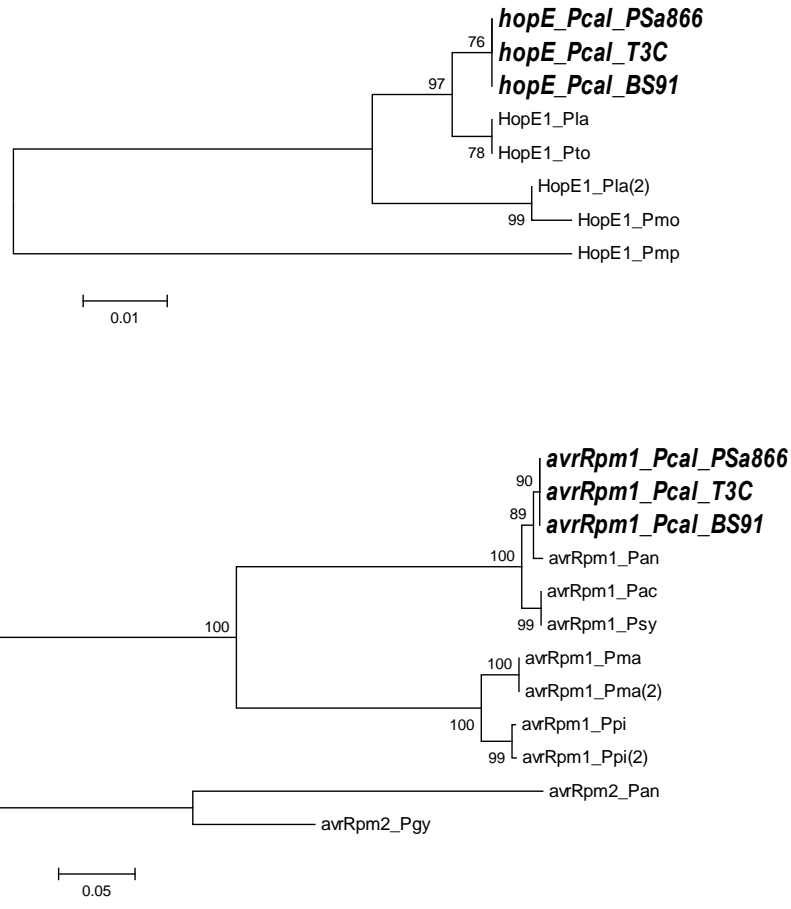

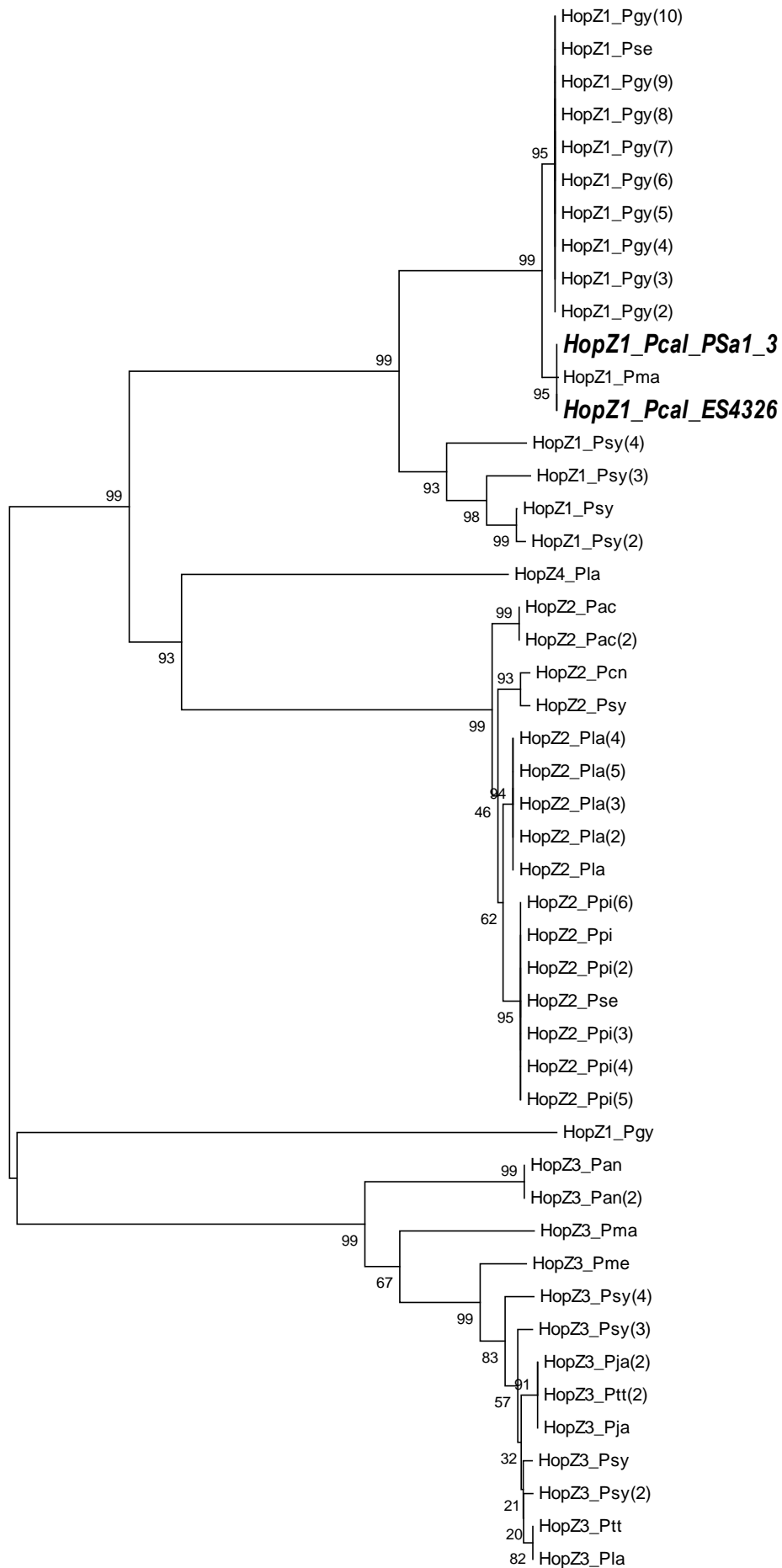

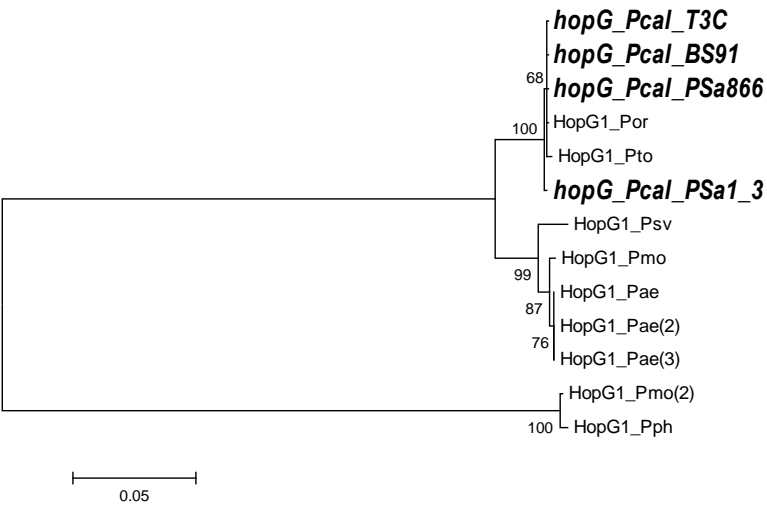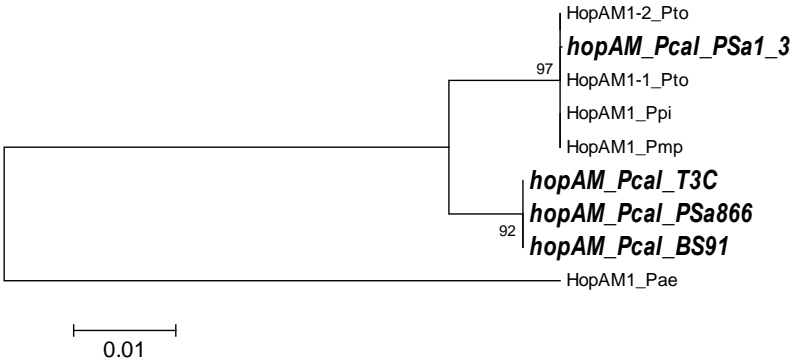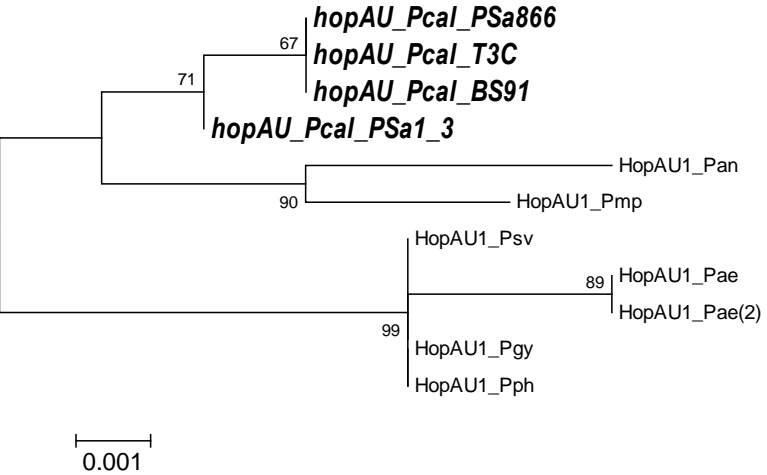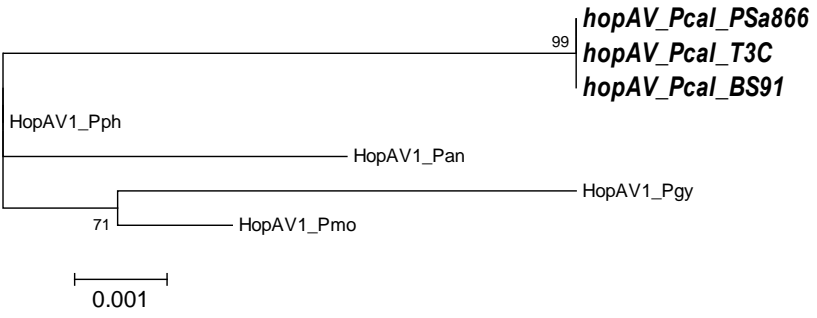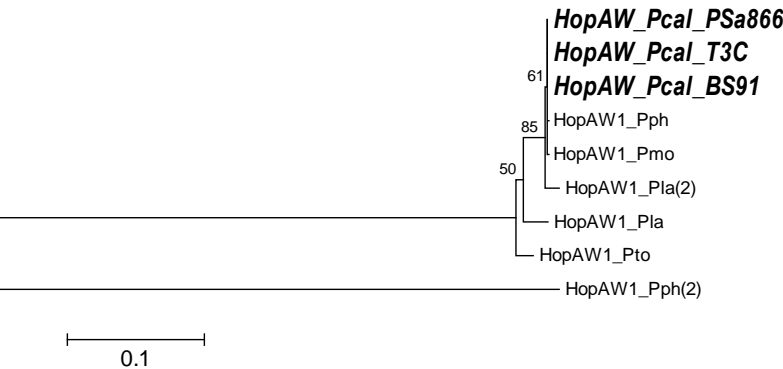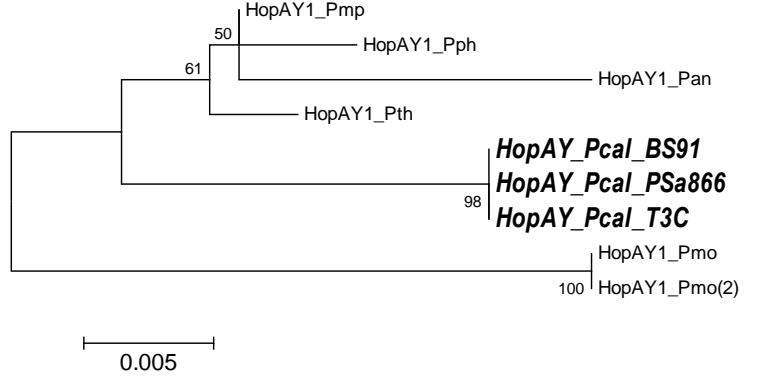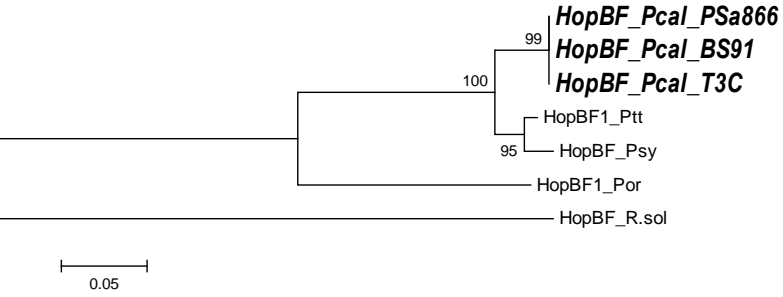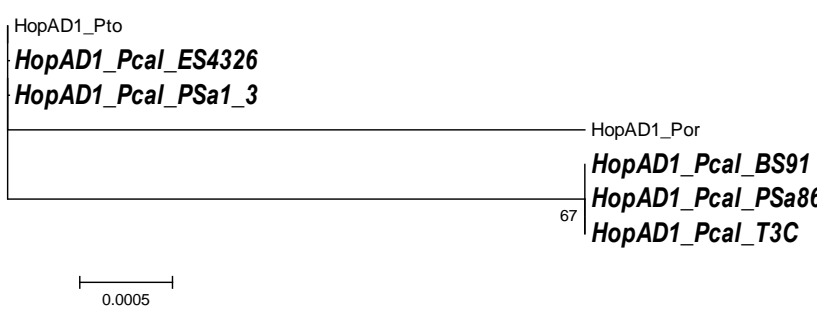

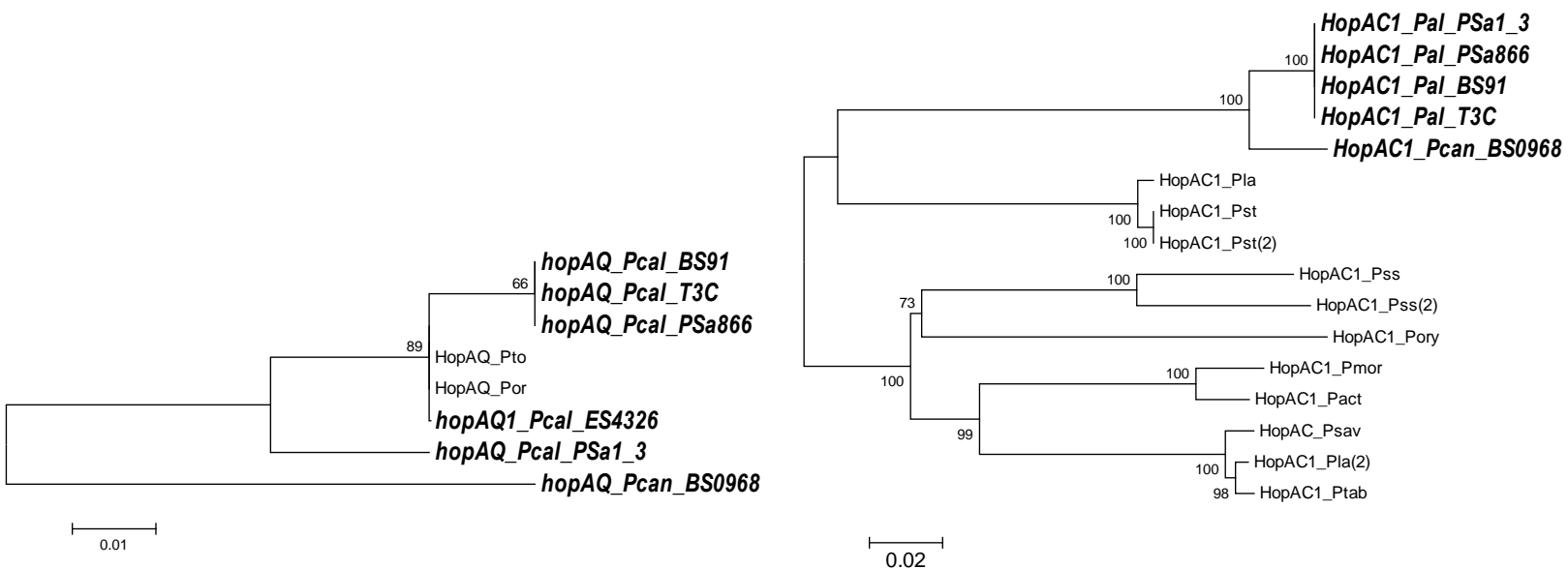

Figure S4

Supplement: Figure S4 — T3SS effector proteins (T3EPs) phylogeny. For the phylogenetic analysis, the amino acid sequences of the Pcal effector as well as of other effectors, as they are presented in the Hop Database website, were used. Additional information for the phylogeny of the rest of Pcal T3EPs can be found in Figure S8. (PDF) [file pone.0059366.s004.pdf]

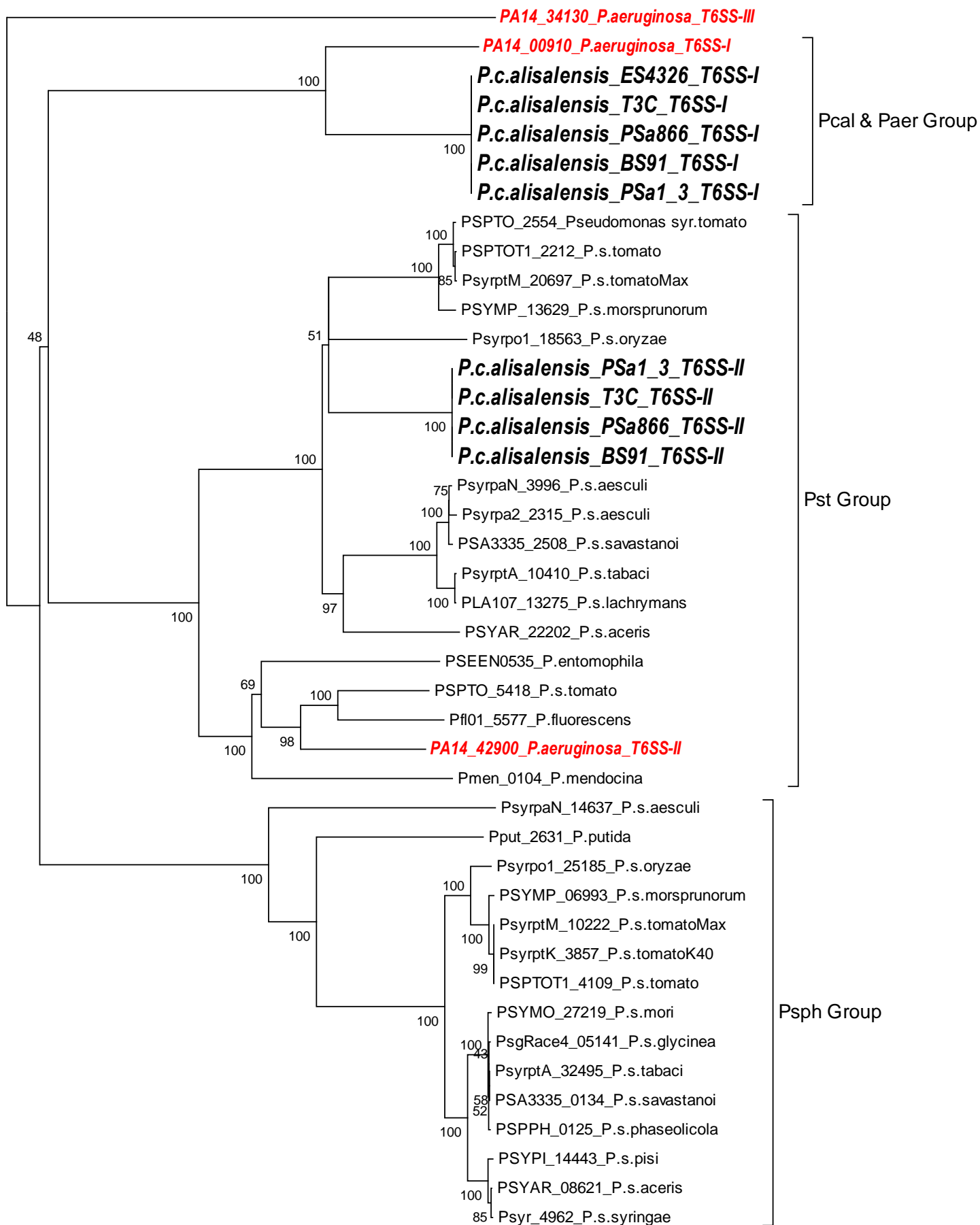

Figure S5

Supplement: Figure S5 — Phylogenetic analysis based on the protein sequences of the T6SS component, ImpL. For the phylogenetic analysis the amino acid sequences were used. Information for additional phylogenetic analysis of various T6SS core components can be found in figure S6. (PDF) [file pone.0059366.s005.pdf]

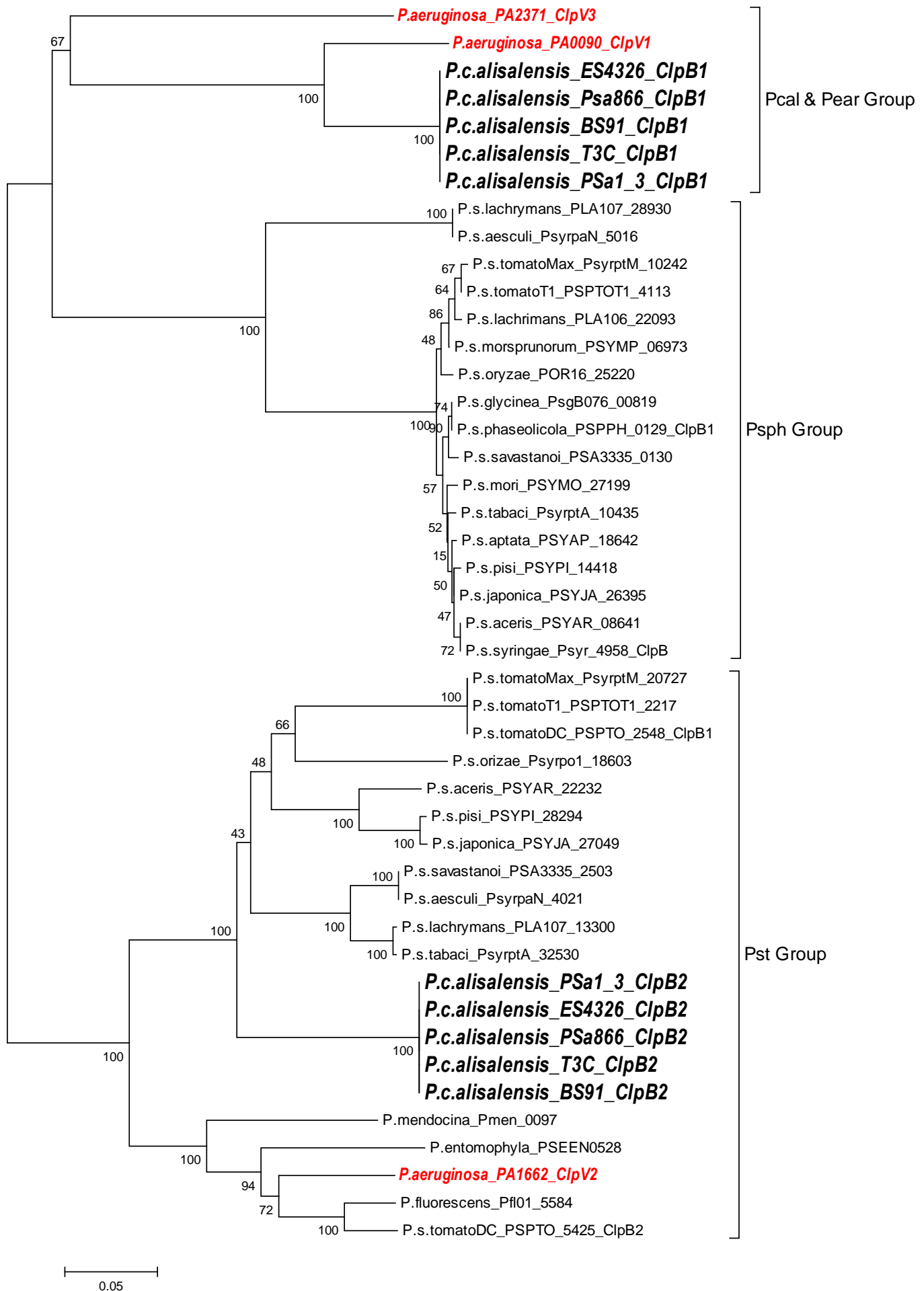

Figure S6

Supplement: Figure S6 — Phylogenetic analysis based on the T6SS protein sequences of the T6SS ATPase, ClpV/B. For the phylogenetic analysis the amino acid sequences were used. Information for additional phylogenetic analysis of various T6SS core components can be found in figure S5. (PDF) [file pone.0059366.s006.pdf]
