# Supplementary material for: Comparative Genomics of Multiple Strains of Pseudomonas cannabina pv. alisalensis, a Potential Model Pathogen of Both Monocots and Dicots
Source: PLoS One. 2013 Mar 28;8(3):e59366. doi: 10.1371/journal.pone.0059366 (PMC3610874; doi:10.1371/journal.pone.0059366)
Supplement: Table S1 — Single nucleotide polymorphisms between Pcal PSa1_3, PSa866 and T3C hrp/hrc clusters. (DOCX) [file pone.0059366.s007.docx]

| **SNP pattern** | **PSa866** | **T3C** | **PSa1_3** |
| --- | --- | --- | --- |
| ttc | 461 | 461 | 461 |
| gga | 823 | 823 | 823 |
| aac | 1592 | 1592 | 1592 |
| gga | 2910 | 2910 | 2910 |
| ggc | 2976 | 2976 | 2976 |
| gga | 3131 | 3131 | 3131 |
| ttc | 3152 | 3152 | 3152 |
| cct | 5829 | 5829 | 5834 |
| nnc | 7482 | 7482 | 7487 |
| cct | 9873 | 9873 | 9875 |
| ccg | 10213 | 10213 | 10215 |
| ggc | 10235 | 10235 | 10237 |
| ggt | 10757 | 10757 | 10759 |
| ttc | 11639 | 11639 | 11641 |
| ggt | 11961 | 11961 | 11963 |
| ttc | 12803 | 12803 | 12805 |
| aag | 13173 | 13173 | 13175 |
| ggc | 13360 | 13360 | 13362 |
| aag | 14040 | 14040 | 14042 |
| ttc | 16543 | 16543 | 16544 |
| cca | 16936 | 16936 | 16937 |
| cct | 18552 | 18552 | 18553 |
| cct | 21048 | 21048 | 21048 |
| aag | 21538 | 21538 | 21538 |
| gga | 26159 | 26159 | 26159 |
| aag | 28774 | 28774 | 28774 |
| ttc | 31270 | 31270 | 31270 |
| nng | 32079 | 32079 | 32079 |
| ttc | 32178 | 32178 | 32179 |
| cca | 34651 | 34651 | 34652 |
| cct | 38064 | 38064 | 38065 |
| aag | 40251 | 40251 | 40251 |
| ttg | 40261 | 40261 | 40261 |

**Table S1**: Single nucleotide polymorphisms between the *Pcal* PSa1_3, PSa866 and T3C *hrp*/*hrc* clusters
