# Supplementary material for: Comparative Genomics of Multiple Strains of Pseudomonas cannabina pv. alisalensis, a Potential Model Pathogen of Both Monocots and Dicots
Source: PLoS One. 2013 Mar 28;8(3):e59366. doi: 10.1371/journal.pone.0059366 (PMC3610874; doi:10.1371/journal.pone.0059366)
Supplement: Table S2 — Plasmids present in Pcal strains. (DOCX) [file pone.0059366.s008.docx]

**Table S2**: Plasmids present in *Pcal* strains.

|  | **Strains** | | | | | |
| --- | --- | --- | --- | --- | --- | --- |
| **Plasmids** | **ES4326** | **PSa1_3** | **PSa866** | **T3C** | **BS91** | **BS0968** |
| *pES4326A* | + | + | + | + | + | + |
| *pES4326B* | + | + | + | + | + | + |
| *pES4326C* | + | - | - | - | - | + |
| *pES4326D* | + | - | - | - | - | - |
| *pES4326E* | + | - | - | - | - | - |
